# Supplementary material for: Effect of glucagon-like peptide-1 receptor agonists on glycemic control, and weight reduction in adults: A multivariate meta-analysis
Source: PLoS One. 2023 Jan 25;18(1):e0278685. doi: 10.1371/journal.pone.0278685 (PMC9876280; doi:10.1371/journal.pone.0278685)
Supplement: S1 File — (DOCX) [file pone.0278685.s002.docx]

**S1 File. Details of data extraction in the study.**

The outcomes corresponding to glycemic changes were usually differences in HbA1c levels, changes in fasting plasma glucose levels, changes in self-monitored blood glucose levels from baseline, differences between groups, rate of achievement of an HbA1c target of 6.5% or 7.0%, or other measurements of insulin level or homeostatic model assessment scores among different studies. The outcomes of anthropometric changes were usually differences in body weight, body mass index (BMI), or waist circumference from baseline, differences between groups, or the rate of achievement of a 5%–10% reduction in body weight or BMI. To avoid unit-of-analysis errors,[1] we only extracted the most commonly reported, estimated treatment difference in HbA1c level and body weight from the baseline corresponding to a full dose of GLP-1 Ras in each RCT. The estimated treatment difference was determined using the Revman calculator with the numbers in each arm and the p-values.

1. Cochrane Handbook for Systematic Reviews of Interventions. 5.1.0 ed2011.
