# Supplementary material for: Effect of glucagon-like peptide-1 receptor agonists on glycemic control, and weight reduction in adults: A multivariate meta-analysis
Source: PLoS One. 2023 Jan 25;18(1):e0278685. doi: 10.1371/journal.pone.0278685 (PMC9876280; doi:10.1371/journal.pone.0278685)
Supplement: S1 Table — (PDF) [file pone.0278685.s003.pdf]

S1 Table. Search strategy

Keywords used in **Pubmed**	
Intervention	“Glucagon-Like Peptide 1” OR “Glucagon Like Peptide 1” OR “GLP-1” OR “GLP 1” OR “Glucagon-Like Peptide-1” OR “Glucagon-Like Peptide-1 Receptor” OR “Glucagon Like Peptide 1 Receptor” OR “Peptide-1 Receptor, Glucagon-Like” OR “Receptor, Glucagon-Like Peptide-1” OR “GLP-1R Receptor” OR “GLP 1R Receptor” OR “Receptor, GLP-1R” OR “GLP1R Protein” OR “Protein, GLP1R” OR “GLP-1 Receptor” OR “GLP 1 Receptor” OR “Receptor, GLP-1” OR “GLP1R Receptor” OR “Receptor, GLP1R
Comparison	Placebo
Outcome	“Body Weights” OR “Weight, Body” OR “Weights, Body” OR “Glucose” OR “Hemoglobin A, Glycated” OR “Hb A1a+b” OR “Hb A1c” OR “HbA1” OR “Glycosylated Hemoglobin A” OR “Hemoglobin A, Glycosylated” OR “Hb A1” OR “Glycohemoglobin A” OR “Hemoglobin A(1) ” OR “Hb A1a-2” OR “Hemoglobin, Glycated A1a-2” OR “A1a-2 Hemoglobin, Glycated” OR “Glycated A1a-2 Hemoglobin” OR “Hemoglobin, Glycated A1a 2” OR “Hemoglobin, Glycosylated A1a-1” OR “A1a-1 Hemoglobin, Glycosylated” OR “Glycosylated A1a-1 Hemoglobin” OR “Hemoglobin, Glycosylated A1a 1” OR “Hb A1a-1” OR “Hemoglobin, Glycated A1b” OR “A1b Hemoglobin, Glycated” OR “Glycated A1b Hemoglobin” OR “Hb A1b” OR “Hemoglobin, Glycosylated A1b” OR “A1b Hemoglobin, Glycosylated” OR “Glycosylated A1b Hemoglobin” OR “Glycated Hemoglobin A1c” OR “Hemoglobin A1c, Glycated” OR “Glycosylated Hemoglobin A1c” OR “Hemoglobin A1c, Glycosylated” OR “Glycated Hemoglobins” OR “Hemoglobins, Glycated” OR “Hemoglobin, Glycosylated” OR “Glycosylated Hemoglobin” OR “Glycated Hemoglobin” OR “Hemoglobin, Glycated”
Study type	“Clinical Trials, Randomized” OR “Trials, Randomized Clinical” OR “Controlled Clinical Trials, Randomized”
Keywords used in **Embase**	

Intervention	“GLP 1 agonist ” OR “GLP 1 receptor agonist” OR “glucagon like peptide 1 agonist” OR “glucagon like peptide 1 receptor stimulating agent” OR “long acting GLP 1 agonist” OR “long acting GLP 1 receptor agonist” OR “long acting glucagon like peptide 1 agonist” OR “long acting glucagon like peptide 1 receptor agonist
Comparison	Placebo
Outcome	“total body weight” OR “weight, body” OR “glycated haemoglobin A1c” OR “glycated hemoglobin A1c” OR “glycosylated haemoglobin A1c” OR “glycosylated hemoglobin A1c” OR “haemoglobin a 1c” OR “haemoglobin a (1c) ” OR “haemoglobin A1c” OR “haemoglobin aic” OR “hb a (1c) ” OR “Hb A1c” OR “hba 1c” OR “hba1c” OR “hemoglobin a 1c” OR “hemoglobin a (1c) ” OR “hemoglobin aic
Study type	“controlled trial, randomized” OR “randomised controlled study” OR “randomised controlled trial” OR “randomized controlled study” OR “trial, randomized controlled
Keywords used in **ClinicalTrials.gov**	
Intervention	“Liraglutide”, “Exenatide once weekly”, “Dulaglutide”, “Albiglutide” or “Semaglutide
Study type	Phase 3 or phase 4 interventional study,
