# Supplementary material for: Effect of glucagon-like peptide-1 receptor agonists on glycemic control, and weight reduction in adults: A multivariate meta-analysis
Source: PLoS One. 2023 Jan 25;18(1):e0278685. doi: 10.1371/journal.pone.0278685 (PMC9876280; doi:10.1371/journal.pone.0278685)
Supplement: S2 Table — (PDF) [file pone.0278685.s004.pdf]

S2 Table. Characteristics of included randomized double-blind placebo-controlled studies

Study, design, participants	Study duration [a] (week)	Study arms	n	Mean age (years)	Women proportion, n (%)	Baseline BMI (kg/m²)	Baseline HbA1c (%)	Mean DM duration (years)	Primary and secondary outcomes
Davies, 2021[1]. STEP 2 Phase 3 trial. Obesity/ overweight type 2 DM	68	Semaglutide 1.0 mg sc qw	404	55	203 (50.4)	35.3 (5.9)	8.1 (0.8)	7.7 (5.9)	(1) BW change, achieve goal rate<br>(2) HbA1C, FPG, serum insulin, BMI, WC change, achieve goal rate[d], BP, HR, lipids profiles, hsCRP, PAI-1, calcitonin, amylase, lipase, QoL, AE
		Semaglutide 2.4 mg sc qw	403	56	223 (55.2)	35.9 (6.4)	8.1 (0.8)	8.2 (6.2)	
		Placebo	403	55	190 (47.1)	35.9 (6.5)	8.1 (0.8)	8.2 (6.2)	
Gudbergsen, 2021[2]. LOSEIT Phase 4 trial. Osteoarthritis /Obesity	52	Liraglutide 3 mg/day sc	80	59.2 (10.8)	52 (65)	32.8 (5.5)	NR	NA	(1) BW change, KOOS pain subscale<br>(2) BMI, WC, waist/hip circumference ratio change, KOOS, ICOAP, WOMAC
		Placebo	76	59.3 (9.7)	49 (64)	31.3 (4.0)	NR	NA	
Neeland, 2021[3]. Phase 4 trial. Obesity/overweight	40	Liraglutide 3.0 mg/day sc qd	73	49.6 (9.8)	67 (92%)	37.2 (6.0)	NR	NA	(1) VAT reducing percentage<br>(2) VAT volume, BW, WC, body adipose tissue, liver fat, lean body volume, muscle volume, FBG, insulin change
		Placebo	55	50.9 (8.8)	51 (93%)	38.1 (6.1)	NR	NA	
Wadden, 2021[4].	68	Semaglutide 2.4 mg sc qw	407	46	315 (77.4)	38.1 (6.7)	5.7 (0.3)	NA	(1) BW change, achieve goal rate

(STEP 3 Phase 3 trial). Obesity/over weight		Placebo	204	46	180 (88.2)	37.8 (6.9)	5.8 (0.3)		(2) HbA1C, FPG, fasting serum insulin, BW, BMI, WC change, achieve goal rate, BP, HR, lipids profiles, hsCRP, PAI-1, calcitonin, amylase, lipase, QoL, AE
Wilding, 2021[5]. STEP 1 Phase 3 trial. Obesity/over weight	68	Semaglutide 2.4 mg sc qw	1,306	46 (13)	955 (73.1)	37.8 (6.7)	5.7 (0.3)	NA	(1) BW change, achieve goal rate<br>(2) HbA1C, FPG change, achieve goal rate, fasting insulin, WC change, achieve goal rate, fat mass, lean body mass, BP, HR, lipid profiles, hsCRP, PAI-1, calcitonin, soluble leptin receptor, leptin, aminotransferase ratio, QoL, AE
		Placebo	655	47 (12)	498 (76.0)	38.0 (6.5)	5.7 (0.3)		
Garvey, 2020[6]. SCALE™ Insulin Phase 3 trial. Obesity/over weight type 2 DM with basal insulin	56	Liraglutide 3.0 mg/day sc qd	198	55.9	108 (54.5)	35.9 (6.5)	7.9 (1.1)	11.4 (6.8)	(1) BW change, achieve goal rate<br>(2) HbA1C, FPG, SMBG change, achieve goal rate, total daily insulin dose, WC change, achieve goal rate, BP, HR, lipid profiles, EKG, hematology, biochemistry changes, QoL, AE
		Placebo	198	57.6	99 (50.0)	35.3 (5.8)	8.0 (1.0)	12.8 (6.9)	
Ghanim, 2020[7]. Phase 3 trial. Overweight, type 1 DM	26	Liraglutide 1.8 mg/day sc qd	37	47 (2)	20 (65)	33.3 (1.2)	8.0 (0.2)	NR	(1) HbA1C<br>(2) glucose at week 26
		Placebo	27	45 (3)	16 (59)	29.5 (1.3)	7.8 (0.2)		

Aroda, 2019[8]. PIONEER 1 Phase 3 trial. Type 2 DM treated with diet and exercise only	26	Semaglutide 3 mg oral qd	175	55 (11)	86 (49.1)	31.8 (6.3)	7.9 (0.7)	3.8 (5.3)	(1) HbA1C change<br>(2) BMI, WC, FPG, SMBG change, achieve goal rate, insulin, pro-insulin, glucagon, HOMA, time to rescue medication, anti-Semaglutide antibodies<br>(3) BP, HR, lipid profiles, Amylase, Lipase, CRP, changes, AE, QoL
		Semaglutide 7 mg oral qd	175	56 (11)	82 (46.9)	31.6 (6.4)	8.0 (0.6)	3.6 (5.1)	
		Semaglutide 14 mg oral qd	175	54 (11)	89 (50.9)	31.7 (6.6)	8.0 (0.7)	3.4 (4.4)	
		Placebo	178	54 (11)	89 (50.0)	32.2 (6.9)	7.9 (0.7)	3.4 (4.6)	
Gerstein, 2019[9]. REWIND Phase 3 trial. Type 2 DM, CVD	Median 64.8	Dulaglutide 1.5mg sc qw	4,949	66.2 (6.5)	2,306 (46.6)	32.3 (5.7)	7.3 (1.1)	10.5 (7.3)	(1) MACE, MI, stroke, CV-death<br>(2) Angina, HF, microvascular
		Placebo	4,952	66.2 (6.5)	2,283 (46.1)	32.3 (5.8)	7.4 (1.1)	10.6 (7.2)	
Husain, 2019[10] PIONEER-6 Phase 3 trial. Type 2 DM, high CVD risk	Median 15.9 months	Semaglutide 14 mg oral qd	1,591	66 (7)	507 (31.9)	32.3 (6.6)	8.2 (1.6)	14.7 (8.5)	(1) MACE<br>(2) MI, stroke, death, angina, HF, microvascular, HbA1C, BW change, BP, HR, lipid profiles, AE
		Placebo	1,592	66 (7)	500 (31.4)	32.3 (6.4)	8.2 (1.6)	15.1 (8.5)	
Mosenzonl, 2019[11]. PIONEER 5 Phase 3 trial.	26	Semaglutide 14 mg oral qd	163	71 (8)	80 (49)	32.2 (5.4)	8.0 (0.7)	14.1 (8.6)	(1) HbA1C change<br>(2) FPG change, achieve goal rate, time to rescue medication, anti-semaglutide antibodies, BW, BMI, WC change, achieve goal rate, BP, HR, UACR, ECG, CRP, lipid
		Placebo	161	70 (8)	88 (55)	32.6 (5.5)	7.9 (0.7)	13.9 (7.4)	

Type 2 DM, CKD									profiles, amylase, lipase, AE, QoL
van Eyk, 2019[12] MAGNA VICTORIA Phase 4 trial. Type 2 DM	26	Liraglutide 1.8 mg/day sc qd	22	55 (11)	14 (64)	30.4 (3.8)	8.1 (0.9)	19 (10)	(1) Stroke volume, ejection fraction, cardiac output and index, peak ejection rate (2) Aorta, carotid vessel wall imaging, adipose tissue distribution, total body fat, epicardial fat volume, HbA1c, FBG, BW, BMI change
		Placebo	25	55 (9)	14 (56)	28.6 (4.0)	8.6 (1.1)	17 (10)	
Zinman, 2019[13] SUSTAIN 9 Phase 3 trial. Type 2 DM	30	Semaglutide 1.0 mg sc qw	151	57.5 (8.9)	62 (41.1)	31.1 (6.2)	8.0 (0.8)	9.8 (6.3)	(1) HbA1C change (2) BW, FPG, SMPG, lipid, BMI, WC, hematology, biochemistry, calcitonin, EKG, HR, PE changes, PRO, AE
		Placebo	151	56.6 (10.1)	64 (42.4)	32.7 (6.9)	8.1 (0.8)	9.6 (5.9)	
Zinman, 2019[14] PIONEER 8 Phase 3 trial. Type 2 DM treated with insulin	26	Semaglutide 3 mg oral qd	184	61 (9)	82 (44.6)	31.0 (6.8)	8.2 (0.7)	15.1 (7.9)	(1) HbA1C change (2) FPG, SMBG, achieve goal rate, time to rescue medication, BW, BMI, WC change, achieve goal rate, BP, HR, amylase, lipase, lipid profiles, EKG changes, AE, QoL
		Semaglutide 7 mg oral qd	182	60 (10)	79 (43.4)	31.1 (7.0)	8.2 (0.7)	16.2 (8.6)	
		Semaglutide 14 mg oral qd	181	61 (10)	96 (53.0)	30.8 (6.3)	8.2 (0.7)	14.1 (8.0)	
		Placebo	184	60 (10)	79 (42.9)	31.0 (6.5)	8.2 (0.7)	14.8 (7.9)	
Frøssing, 2018[15].	26	Liraglutide 1.8 mg/day sc qd	48	31.4 (24.6-35.6)	48 (100)	33.3 (5.1)	NR	NA	(1) Endogenous thrombin potential change (2) PAI-1, adrenomedullin, atrial natriuretic peptide, copeptin, hsCRP, bleeding ratio,

LIPT Phase 4 trial. PCOS		Placebo	24	26.2 (24.8-31.5)	24 (100)	33.3 (4.6)	NR	NA	percent liver fat, body composition, anti-müllerian hormone, ovarian volume change, BW
Rodbard, 2018[16] SUSTAIN™ 5 Phase 3 trial. Type 2 DM treated with insulin	30	Semaglutide 0.5 mg sc qw	132	59.1 (28-84)	58 (43.9)	32.8 (21.1-51.4)	8.4 (7.0-10.3)	12.9 (0.4-37.1)	(1) HbA1C change<br>(2) SMBG, FPG change, achieve goal rate, insulin dose, BW change, BP, AE, satisfaction questionnaire
		Semaglutide 1 mg sc qw	131	58.5 (33-80)	54 (41.2)	32.0 (19.5-51.6)	8.3 (6.9-10.8)	13.7 (0.6-36.9)	
		Placebo qw	133	58.8 (19-86)	62 (46.6)	31.8 (21.0-48.8)	8.4 (6.8-11.1)	13.3 (0.8-39.6)	
Ishøy, 2017[17]. TAO Phase 3 trial. Obese, non-DM, schizophrenia	3 months	Exenatide 2 mg sc qw	20	37.4 (10.7)	9 (45)	39.5 (3.5)	5.2 (2.5)	NA	(1) BW change<br>(2) Body fat composition
		Placebo qw	20	34.4 (10.6)	11 (55)	38.6 (6.3)	5.3 (2.6)		
Mensberg, 2017[18]. Phase 4 trial. Type 2 DM.	16	Liraglutide 1.8 mg/day sc qd	17	56.5 (9)	4 (24)	32.5 (3.7)	8.2 (1.4)	6.0 (5.2)	(1) HbA1C change<br>(2) Glycemic control parameters, meal test, BW change, VO2peak, BP, EKG
		Placebo	16	55.6 (12)	6 (38)	32.4 (5.2)	8.0 (1.2)	3.7 (3.3)	
Sorli, 2017[19]	30	Semaglutide 0.5 mg sc qw	128	54.6 (11.1)	68 (53)	32.5 (7.6)	8.1 (0.9)	4.8 (6.1)	(1) HbA1C change<br>(2) FPG change, achieve goal rate, BW, WC

SUSTAIN™ 1 Phase 3 trial. Drug-naïve type 2 DM		Semaglutide 1 mg sc qw	130	52.7 (11.9)	50 (38)	33.9 (8.4)	8.1 (0.8)	3.6 (4.9)	change, BP
		Placebo qw	129	53.9 (11.0)	59 (46)	32.4 (6.9)	8.0 (0.9)	4.1 (5.5)	
Blackman, 2016[20]. SCALE™ phase 3 trial. Obesity with OSA	32	liraglutide 3.0 mg/day sc qd	180	48.6 (9.9)	51 (28.3)	38.9 (6.4)	5.7 (0.4)	NA	(1) AHI change<br>(2) BW, FPG, HbA1C change
		Placebo	179	48.4 (9.5)	50 (27.9)	39.4 (7.4)	5.6 (0.4)	NA	
Davies, 2016[21]. Phase 3 trial. Type 2 DM with CKD	26	Liraglutide 1.8 mg/day sc qd	140	68.0 (8.3)	65 (46.4)	33.4 (5.4)	8.1 (0.8)	15.9 (8.9)	(1) HbA1C change<br>(2) SMPG change, achieve goal rate, BMI, eGFR change
		Placebo	137	66.3 (8.0)	72 (52.6)	34.5 (5.4)	8.0 (0.9)	14.2 (7.5)	
Dejgaard, 2016[22]. Phase 4 trial. Overweight type 1 DM	24	Liraglutide 1.8 mg/day sc qd	50	47 (13)	20 (40)	30.3 (3.5)	8.7 (0.7)	20 (12)	(1) HbA1C change<br>(2) Glycemic excursions, BMI change, QoL, CV
		Placebo	50	49 (12)	15 (30)	29.8 (3.1)	8.7 (0.7)	25 (12)	
Kuhadiya, 2016[23]. Phase 3 trial. Type 1 DM	12	Liraglutide 0.6 mg/day sc qd	14	45 (4)	9 (64.3)	26 (3)	7.5 (0.2)	25 (2)	(1) Weekly glucose concentrations change<br>(2) HbA1C, BW, total insulin dose change, AUC of glucose, acetaminophen concentration, carbohydrate intake, Insulin, C-peptide, incretin following meal
		Liraglutide 1.2 mg/day sc qd	16	42 (3)	5 (31.3)	33 (2)	7.8 (0.2)	21 (3)	
		Liraglutide 1.8 mg/day sc qd	16	42 (3)	11 (68.8)	28 (4)	7.4 (0.2)	20 (3)	

		Placebo	17	50 (3)	10 (58.8)	28 (2)	7.7 (0.2)	30 (3)	
Marso, 2016[24] SUSTAIN™Phase 3 trial). Type 2 DM and CVD	104	Semaglutide 0.5 mg sc qw	826	64.6 (7.3)	331 (40.1)	NR	8.7 (1.4)	14.3 (8.2)	(1) MACE (CV death, non-fatal MI, non-fatal stroke)<br>(2) CV outcome, death, H bA1C, BW, FPG, lipid profiles, UACR change, BP, HR, lipid, AE, anti-semaglutide antibodies, PRO
		Semaglutide 1 mg sc qw	822	64.7 (7.1)	304 (37.0)		8.7 (1.5)	14.1(8.2)	
		Placebo 0.5 mg sc qw	824	64.8 (7.6)	342 (41.5)		8.7 (1.5)	14.0 (8.5)	
		Placebo 1 mg sc qw	825	64.4 (7.5)	318 (38.5)		8.7 (1.5)	13.2 (7.4)	
Nauck, 2016[25]. HARMONY 2 Phase 3 trial. Type 2 DM	52	Albiglutide 30 mg sc qw	101	53.6 (10.9)	43 (42.6)	33.7 (5.1)	8.0 (0.8)	3.4 (3.7)	(1) HbA1C change<br>(2) Time to hyperglycemia rescue, FPG, BW, postprandial glucose change, achieve goal rate, Albiglutide concentration
		Albiglutide 50 mg sc qw	99	52.0 (11.8)	49 (49.5)	33.9 (5.5)	8.2 (0.9)	4.2 (4.6)	
		Placebo	101	53.1 (11.7)	43 (42.6)	33.0 (5.4)	8.0 (0.9)	4.3 (4.0)	
Vanderheiden, 2016[26]. Phase 4 trial. Type 2 DM/ obesity	6 months	Liraglutide 1.8mg/day sc qd	35	52.8 (8.1)	23 (66)	40.7 (6.7)	9.0 (1.2)	16 (12-23)	(1) HbA1c change<br>(2) TG content, BW change, Beta-Cell function, glucagon, insulin dose, number of daily injection, BP, lipid profiles, ae, liver function, QoL, Beta-cell function
		Placebo	36	55.5 (6.6)	22 (61)	41.6 (10.4)	8.9 (1.0)	18 (13-27)	
Ahmann, 2015[27]. Phase 3 trial. Type 2 DM with basal insulin	26	Liraglutide 1.8mg/day sc qd	225	59.3 (9.2)	105 (46.7)	32.3 (5.6)	8.2 (0.8)	12.1 (7.1)	(1) HbA1C change<br>(2) FPG, SMBG, BW change, achieve goal rate, AE
		Placebo	225	57.5 (11.1)	89 (39.6)	32.2 (5.7)	8.3 (0.9)	12.1 (6.8)	

Davies 2015[28] . SCALE™ phase 3 trial. Obesity/ overweight type 2 DM	56	Liraglutide 3.0mg/day sc qd	423	55.0 (10.8)	203 (48.0)	37.1 (6.5)	7.9 (0.8)	7.5 (5.7)	(1) BW change<br>(2) HbA1C, achieve goal rate, WC, BW changes, hypoglycemia
		Liraglutide 1.8mg/day sc qd	211	54.9 (10.7)	103 (48.8)	37.0 (6.9)	8.0 (0.8)	7.4 (5.2)	
		Placebo	212	54.7 (9.8)	103 (54.2)	37.4 (7.1)	7.9 (0.8)	6.7 (5.1)	
Retnakaran, 2014[29]. LIBRA Phase 3 trial. Type 2 DM	48	Liraglutide 6.0 mg/mL	26	58.9 (8.7)	10 (38.5)	30.0 (4.3)	6.4 (0.5)	3.0 (2-5)	(1) Insulin Secretion-Sensitivity Index-2<br>(2) HbA1c, FPG change
		Placebo	25	57.4 (7.4)	9 (36)	30.4 (5.8)	6.2 (0.4)	1.5 (0.75-3)	
Kim, 2013[30]. Phase 3 trial. Pre-diabetes	14	Liraglutide 1.8mg/day sc qd	24	58 (7)	16 (67)	31.9 (2.7)	NR	NA	(1) BW change<br>(2) Insulin AUC, insulin resistance after calorie restriction
		Placebo	27	58 (8)	17 (63)	31.9 (3.5)			
Wadden, 2013[31]. SCALE Maintenance phase 3 trial. Obese/ overweight	56	Liraglutide 3.0mg/day sc qd	212	45.9 (11.9)	84 (39.6)	36.0 (5.9)	5.6 (0.4)	NA	(1) BW change, achieve goal rate<br>(2) BP, HR, lipid profiles, CVD marker, metabolic syndrome criteria, WC, BMI changes, glycemia control parameters
		Placebo	210	46.5 (11)	79 (37.6)	35.2 (5.9)	5.5 (0.4)	NA	

Continuous variables were presented with mean (standard deviation or range), categorial variables were presented with number (percentage)

[a] Study period referred to the period of primary outcome assessed

[b] Achieve goal rate in weight control referred to weight reduction >5%, 10, 20 % depending on studies

[b] Achieve goal rate in diabetes control referred to glycated hemoglobin ≦7 or 6.5 % depending on studies

Abbreviations: AE: Adverse events; AHI: Apnea-hypopnea index; AUC: Area under curve; AWARD-8*:* Study of How Dulaglutide Compares to Placebo in Participants With Type 2 Diabetes Who Are Also on Sulfonylurea Therapy; BMI: Body mass index; BP: Blood pressure; BW: Body weight; CKD: Chronic kidney disease; CRP: C-reactive protein; CV: Cardiovascular; CVD: Cardiovascular disease; DM, Diabetes mellitus; eGFR: Estimated Glomerular filtration rate; EKG: Electrocardiogram; FPG: Fasting plasma glucose; HbA1c: Glycated hemoglobin; HF: Heart failure; HOMA: Homeostatic model assessment; HR: Heart rate; hsCRP: High sensitivity C-reactive protein; ICOAP, Intermittent and Constant Osteoarthritis Pain questionnaire; KKOOS, Knee injury and Osteoarthritis Outcome Score; LIBRA, LIraglutide and Beta-cell RepAir; LIPT, Liraglutide in Polycystic Ovary Syndrome; LOSEIT, Liraglutide 3 mg for Knee Osteoarthritis; MACE: Major adverse cardiac events; MAGNA VICTORIA, Effect of Liraglutide on Cardiovascular Endpoints in Diabetes Mellitus Type 2 Patients of South Asian Descent; MI: Myocardial infarction; NA: Not-applicable; NR: Non-reported; OSA: Obstructive sleep apnea; PAI-1: Plasminogen activator inhibitor-1 activity; PIONEER 1: Efficacy and Safety of Oral Semaglutide Versus Placebo in Subjects With Type 2 Diabetes Mellitus Treated With Diet and Exercise Only; PIONEER 5: Efficacy and Safety of Oral Semaglutide Versus Placebo in Subjects With Type 2 Diabetes and Moderate Renal Impairment; PIONEER-6: A Trial Investigating the Cardiovascular Safety of Oral Semaglutide in Subjects With Type 2 Diabetes; PIONEER 8: Efficacy and Safety of Oral Semaglutide Versus Placebo in Subjects With Type 2 Diabetes Mellitus Treated With Insulin; PRO: Patient reported outcome; REWIND: Researching Cardiovascular Events With a Weekly Incretin in Diabetes; sc: Subcutaneous; SMBG: Self-monitoring of blood glucose; STEP 1: Research Study Investigating How Well Semaglutide Works in People Suffering From Overweight or Obesity; STEP 2: Research Study Investigating How Well Semaglutide Works in People With Type 2 Diabetes Suffering From Overweight or Obesity; STEP 3: Research Study to Look at How Well Semaglutide is at Lowering Weight When Taken Together With an Intensive Lifestyle Program; SUSTAIN™ 1: Efficacy and Safety of Semaglutide Once-weekly Versus Placebo in Drug-naïve Subjects With Type 2 Diabetes; SUSTAIN™ 5: Efficacy and Safety of Semaglutide Once-weekly Versus Placebo as add-on to Basal Insulin Alone or Basal Insulin in Combination With Metformin in Subjects With Type 2 Diabetes; SUSTAIN™ 6: Trial to Evaluate Cardiovascular and Other Long-term Outcomes With Semaglutide in Subjects With Type 2 Diabetes; TAO: Treatment of Antipsychotic-associated Obesity With a GLP-1 Analogue; TG: Triglyceride; QoL: Quality of Life; Qw: Once per week; UACR: Urinary albumin to creatinine ratio; VAT: Visceral adipose tissue; VO2peak: Maximal oxygen uptake; WOMAC, Western Ontario and McMaster Universities Arthritis Index; WC: Waist circumference

Reference

1. Davies M, Færch L, Jeppesen OK, Pakseresht A, Pedersen SD, Perreault L, et al. Semaglutide 2·4 mg once a week in adults with overweight or obesity, and type 2 diabetes (STEP 2): a randomised, double-blind, double-dummy, placebo-controlled, phase 3 trial. The Lancet. 2021;397(10278):971-84. doi: 10.1016/S0140-6736(21)00213-0.

2. Gudbergsen H, Overgaard A, Henriksen M, Wæhrens EE, Bliddal H, Christensen R, et al. Liraglutide after diet-induced weight loss for pain and weight control in knee osteoarthritis: a randomized controlled trial. The American journal of clinical nutrition. 2021;113(2):314-23. Epub 2021/01/21. doi: 10.1093/ajcn/nqaa328. PubMed PMID: 33471039.

3. Neeland IJ, Marso SP, Ayers CR, Lewis B, Oslica R, Francis W, et al. Effects of liraglutide on visceral and ectopic fat in adults with overweight and obesity at high cardiovascular risk: a randomised, double-blind, placebo-controlled, clinical trial. The lancet Diabetes & endocrinology. 2021;9(9):595-605. Epub 2021/08/07. doi: 10.1016/s2213-8587(21)00179-0. PubMed PMID: 34358471.

4. Wadden TA, Bailey TS, Billings LK, Davies M, Frias JP, Koroleva A, et al. Effect of Subcutaneous Semaglutide vs Placebo as an Adjunct to Intensive Behavioral Therapy on Body Weight in Adults With Overweight or Obesity: The STEP 3 Randomized Clinical Trial. Jama. 2021;325(14):1403-13. Epub 2021/02/25. doi: 10.1001/jama.2021.1831. PubMed PMID: 33625476; PubMed Central PMCID: PMCPMC7905697 Nordisk (from grant support to the University of Pennsylvania) and personal fees from Novo Nordisk for service on a scientific advisory board during the conduct of the study, as well as personal fees from WW International (formerly Weight Watchers) for service on a scientific advisory board outside the submitted work. Dr Bailey reports receiving grants, personal fees, and nonfinancial support (writing assistance) from Novo Nordisk during the conduct of the study. Dr Billings reports receiving personal fees from Novo Nordisk, Sanofi, and Eli Lilly outside the submitted work. Dr Davies is co-funded by the NIHR Leicester Biomedical Research Centre and reports receiving consulting fees from Novo Nordisk; advisory board member, speaker, and consulting fees from Sanofi-Aventis, Eli Lilly, Merck Sharp & Dohme, Boehringer Ingelheim, AstraZeneca, and Janssen; advisory board fees from Lexicon, Servier, and Gilead Sciences Ltd; speaker fees from Napp Pharmaceuticals; and grants from Novo Nordisk, Sanofi-Aventis, Lilly, Boehringer Ingelheim, AstraZeneca, and Janssen outside the submitted work. Dr Frias reports receiving research support grants from Novo Nordisk during the conduct of the study; grants and personal fees from Boehringer Ingelheim, Eli Lilly, Merck, Novo Nordisk, and Sanofi; and grants from Janssen and Pfizer outside the submitted work. Dr Koroleva reports being an employee of Novo Nordisk A/S and holding shares in the company. Dr Lingvay reports receiving advisory board fees and consulting fees from AstraZeneca, consulting fees from Bayer HealthCare Pharmaceuticals, Eli Lilly and Company, Intarcia, Intercept Pharmaceuticals, Janssen Global Services, MannKind Corporation, Target Pharma, Valeritas, and Zealand Pharma; advisory board fees from Boehringer Ingelheim and Sanofi US Services; grant support, paid to UT Southwestern, from Merck; grant support, paid to her institution, from Mylan Pharmaceuticals and Pfizer; grant support, paid to UT Southwestern; and advisory board fees, consulting fees, and travel support from Novo Nordisk. Dr O'Neil reports receiving grants from Novo Nordisk during the conduct of the study; grants from Epitomee Medical, Eli Lilly, and WW International; and personal fees from Robard, Gedeon Richter, WebMD, and Novo Nordisk outside the submitted work. Dr Rubino reports receiving

writing assistance from Novo Nordisk during the conduct of the study; serving as a clinical investigator for AstraZeneca, Boehringer Ingelheim, and Novo Nordisk; and reports owning Novo Nordisk shares of stock outside the submitted work. Dr Skovgaard reports spousal employment at Novo Nordisk. Dr Wallenstein reports receiving personal fees from Novo Nordisk during the conduct of the study and outside the submitted work. Dr Garvey reports receiving grants from Novo Nordisk; serving as site principal investigator for the clinical trial, which was sponsored by his university during the conduct of the study; receiving grants from Lexicon and Pfizer outside the submitted work; and serving as an ad hoc consultant on advisory committees for Jazz Pharmaceuticals, Boehringer Ingelheim, Novo Nordisk, and Pfizer. In each instance, he received no financial compensation, nor was there a financial relationship. No other disclosures were reported.

5. Wilding JPH, Batterham RL, Calanna S, Davies M, Van Gaal LF, Lingvay I, et al. Once-Weekly Semaglutide in Adults with Overweight or Obesity. 2021;384(11):989-1002. doi: 10.1056/NEJMoa2032183. PubMed PMID: 33567185.

6. Garvey WT, Birkenfeld AL, Dicker D, Mingrone G, Pedersen SD, Satylganova A, et al. Efficacy and Safety of Liraglutide 3.0 mg in Individuals With Overweight or Obesity and Type 2 Diabetes Treated With Basal Insulin: The SCALE Insulin Randomized Controlled Trial. Diabetes care. 2020;43(5):1085-93. Epub 2020/03/07. doi: 10.2337/dc19-1745. PubMed PMID: 32139381; PubMed Central PMCID: PMCPMC7171937.

7. Ghanim H, Batra M, Green K, Abuaysheh S, Hejna J, Makdissi A, et al. Liraglutide treatment in overweight and obese patients with type 1 diabetes: A 26-week randomized controlled trial; mechanisms of weight loss. Diabetes, obesity & metabolism. 2020;22(10):1742-52. Epub 2020/05/20. doi: 10.1111/dom.14090. PubMed PMID: 32424935.

8. Aroda VR, Rosenstock J, Terauchi Y, Altuntas Y, Lalic NM, Morales Villegas EC, et al. PIONEER 1: Randomized Clinical Trial of the Efficacy and Safety of Oral Semaglutide Monotherapy in Comparison With Placebo in Patients With Type 2 Diabetes. Diabetes care. 2019;42(9):1724-32. Epub 2019/06/13. doi: 10.2337/dc19-0749. PubMed PMID: 31186300.

9. Gerstein HC, Colhoun HM, Dagenais GR, Diaz R, Lakshmanan M, Pais P, et al. Dulaglutide and cardiovascular outcomes in type 2 diabetes (REWIND): a double-blind, randomised placebo-controlled trial. Lancet (London, England). 2019;394(10193):121-30. Epub 2019/06/14. doi: 10.1016/s0140-6736(19)31149-3. PubMed PMID: 31189511.

10. Husain M, Birkenfeld AL, Donsmark M, Dungan K, Eliaschewitz FG, Franco DR, et al. Oral Semaglutide and Cardiovascular Outcomes in Patients with Type 2 Diabetes. The New England journal of medicine. 2019;381(9):841-51. Epub 2019/06/12. doi: 10.1056/NEJMoa1901118. PubMed PMID: 31185157.

11. Mosenzon O, Blicher TM, Rosenlund S, Eriksson JW, Heller S, Hels OH, et al. Efficacy and safety of oral semaglutide in patients with type 2 diabetes and moderate renal impairment (PIONEER 5): a placebo-controlled, randomised, phase 3a trial. The lancet Diabetes & endocrinology. 2019;7(7):515-27. Epub 2019/06/14. doi: 10.1016/s2213-8587(19)30192-5. PubMed PMID: 31189517.

12. van Eyk HJ, Paiman EHM, Bizino MB, de Heer P, Geelhoed-Duijvestijn PH, Kharagjitsingh AV, et al. A double-blind, placebo-controlled, randomised trial to assess the effect of liraglutide on ectopic fat accumulation in South Asian type 2 diabetes patients. Cardiovascular diabetology. 2019;18(1):87. Epub

2019/07/11. doi: 10.1186/s12933-019-0890-5. PubMed PMID: 31288820; PubMed Central PMCID: PMCPMC6615254.
13. Zinman B, Bhosekar V, Busch R, Holst I, Ludvik B, Thielke D, et al. Semaglutide once weekly as add-on to SGLT-2 inhibitor therapy in type 2 diabetes (SUSTAIN 9): a randomised, placebo-controlled trial. The lancet Diabetes & endocrinology. 2019;7(5):356-67. Epub 2019/03/06. doi: 10.1016/s2213-8587(19)30066-x. PubMed PMID: 30833170.
14. Zinman B, Aroda VR, Buse JB, Cariou B, Harris SB, Hoff ST, et al. Efficacy, Safety, and Tolerability of Oral Semaglutide Versus Placebo Added to Insulin With or Without Metformin in Patients With Type 2 Diabetes: The PIONEER 8 Trial. Diabetes care. 2019;42(12):2262-71. Epub 2019/09/19. doi: 10.2337/dc19-0898. PubMed PMID: 31530667; PubMed Central PMCID: PMCPMC7364672.
15. Frøssing S, Nylander M, Chabanova E, Frystyk J, Holst JJ, Kistorp C, et al. Effect of liraglutide on ectopic fat in polycystic ovary syndrome: A randomized clinical trial. Diabetes, obesity & metabolism. 2018;20(1):215-8. Epub 2017/07/07. doi: 10.1111/dom.13053. PubMed PMID: 28681988.
16. Rodbard HW, Lingvay I, Reed J, de la Rosa R, Rose L, Sugimoto D, et al. Semaglutide Added to Basal Insulin in Type 2 Diabetes (SUSTAIN 5): A Randomized, Controlled Trial. The Journal of clinical endocrinology and metabolism. 2018;103(6):2291-301. Epub 2018/04/25. doi: 10.1210/jc.2018-00070. PubMed PMID: 29688502; PubMed Central PMCID: PMCPMC5991220.
17. Ishøy PL, Knop FK, Broberg BV, Bak N, Andersen UB, Jørgensen NR, et al. Effect of GLP-1 receptor agonist treatment on body weight in obese antipsychotic-treated patients with schizophrenia: a randomized, placebo-controlled trial. Diabetes, Obesity and Metabolism. 2017;19(2):162-71. doi: 10.1111/dom.12795. PubMed Central PMCID: PMCAstra Zeneca(Sweden).
18. Mensberg P, Nyby S, Jørgensen PG, Storgaard H, Jensen MT, Sivertsen J, et al. Near-normalization of glycaemic control with glucagon-like peptide-1 receptor agonist treatment combined with exercise in patients with type 2 diabetes. Diabetes, Obesity and Metabolism. 2017;19(2):172-80. doi: 10.1111/dom.12797. PubMed Central PMCID: PMCNovo Nordisk.
19. Sorli C, Harashima SI, Tsoukas GM, Unger J, Karsbøl JD, Hansen T, et al. Efficacy and safety of once-weekly semaglutide monotherapy versus placebo in patients with type 2 diabetes (SUSTAIN 1): a double-blind, randomised, placebo-controlled, parallel-group, multinational, multicentre phase 3a trial. The lancet Diabetes & endocrinology. 2017;5(4):251-60. Epub 2017/01/24. doi: 10.1016/s2213-8587(17)30013-x. PubMed PMID: 28110911.
20. Blackman A, Foster GD, Zammit G, Rosenberg R, Aronne L, Wadden T, et al. Effect of liraglutide 3.0 mg in individuals with obesity and moderate or severe obstructive sleep apnea: the SCALE Sleep Apnea randomized clinical trial. International journal of obesity (2005). 2016;40(8):1310-9. Epub 2016/03/24. doi: 10.1038/ijo.2016.52. PubMed PMID: 27005405; PubMed Central PMCID: PMCPMC4973216 Valeant Canada, Paladin Labs Inc. Dr Foster: at the time of the study was a scientific advisory board participant for Con Agra Foods, United Health Group and Tate and Lyle, and a consultant to Eisai and Novo Nordisk; currently employed by Weight Watchers International. Dr Zammit: consultant for Acorda, Actelion, Alexza, Arena, Aventis, Biovail, Boehringer-Ingelheim, Cephalon, Elan, Eli Lilly, Evotec, Forest, Glaxo Smith Kline, Jazz, King Pharmaceuticals, Ligand, McNeil, Merck, Neurocrine Biosciences, Organon, Pfizer, Purdue, Renovis, Sanofi-Aventis, Select Comfort, Sepracor, Shire, Somnus, Takeda Pharmaceuticals, Vela, Wyeth;

grants/research support from Abbott, Abbvie, Actelion, Ancile, Apnex, Arena, Astra-Zeneca, Aventis, Banyu, Biomarin, BMS, Catalyst, Cephalon Inc., CHDI, Elan, Epix, Eisai, Elminda, Evotec, Forest, Galderma, Genentech, Gilead, Glaxo Smith Kline, Gilead, H. Lundbeck A/S, Janssen, Johnson & Johnson, King, Merck and Co., National Institute of Health (NIH), Neurim, Neurocrine Biosciences, Naurex, Neurim, Neurogen, Novo Nordisk, Organon, Orphan Medical, Otsuka, Pfizer, Predix, Respironics, Saladax, Sanofi-Aventis, Sanofi-Synthelabo, Schering-Plough, Sepracor, Sunovion, Shire, Somaxon, Takeda Pharmaceuticals North America, Targacept, Teva, Thymon, Transcept, UCB Pharma, Ultragenyx, Predix, Vanda, Wyeth-Ayerst Research; honoraria received from Neurocrine Biosciences, King Pharmaceuticals, McNeil, Sanofi-Aventis, Sanofi-Synthelabo, Sepracor, Takeda Pharmaceuticals, Vela Pharmaceuticals, Wyeth-Ayerst Research; ownership interest in Clinilabs, Inc., Clinilabs IPA, Inc., Home Sleep and Respiratory Care, Nationwide Sleep Testing. Dr Rosenberg: received research grant support from Merck, Novo Nordisk, Pfizer, Teva, Jazz Pharmaceuticals, Sunovion Pharmaceuticals and Philips-Respironics. Dr Aronne: advisory board member for Myos Corporation; consultant, speaker, advisor, or received research support from Aspire Bariatrics Inc., Eisai, Ethicon Endo-Surgery Inc., GlaxoSmithKline Consumer Healthcare LP, GI Dynamics, Novo Nordisk, Pfizer, Takeda Pharmaceuticals, Vivus Inc., Zafgen Inc.; ownership interest in Myos Corporation and BMIQ. Dr Wadden: advisory board participant for Orexigen Pharmaceuticals, Inc., Novo Nordisk, Nutrisystem, and Shire Pharmaceuticals, with research support (to the University of Pennsylvania) from the first three companies. Dr Claudius: employee of Novo Nordisk and owns company stock. Dr Jensen: employee of Novo Nordisk and owns company stock. Dr Mignot: advisory board member, consultant for and research support from Jazz Pharmaceuticals; consultant for and research grant from Novo Nordisk; consultant for Reset Pharmaceuticals and Merck; expert witness for FTC.

21. Davies MJ, Bain SC, Atkin SL, Rossing P, Scott D, Shamkhalova MS, et al. Efficacy and Safety of Liraglutide Versus Placebo as Add-on to Glucose-Lowering Therapy in Patients With Type 2 Diabetes and Moderate Renal Impairment (LIRA-RENAL): A Randomized Clinical Trial. Diabetes care. 2016;39(2):222-30. Epub 2015/12/19. doi: 10.2337/dc14-2883. PubMed PMID: 26681713.

22. Dejgaard TF, Frandsen CS, Hansen TS, Almdal T, Urhammer S, Pedersen-Bjergaard U, et al. Efficacy and safety of liraglutide for overweight adult patients with type 1 diabetes and insufficient glycaemic control (Lira-1): A randomised, double-blind, placebo-controlled trial. The Lancet Diabetes and Endocrinology. 2016;4(3):221-32. doi: 10.1016/S2213-8587(15)00436-2. PubMed Central PMCID: PMCNovo Nordisk(Denmark).

23. Kuhadiya ND, Dhindsa S, Ghanim H, Mehta A, Makdissi A, Batra M, et al. Addition of Liraglutide to Insulin in Patients With Type 1 Diabetes: A Randomized Placebo-Controlled Clinical Trial of 12 Weeks. Diabetes care. 2016;39(6):1027-35. Epub 2016/05/22. doi: 10.2337/dc15-1136. PubMed PMID: 27208343; PubMed Central PMCID: PMCPMC5864130.

24. Marso SP, Bain SC, Consoli A, Eliaschewitz FG, Jódar E, Leiter LA, et al. Semaglutide and Cardiovascular Outcomes in Patients with Type 2 Diabetes. 2016;375(19):1834-44. doi: 10.1056/NEJMoa1607141. PubMed PMID: 27633186.

25. Nauck MA, Stewart MW, Perkins C, Jones-Leone A, Yang F, Perry C, et al. Efficacy and safety of once-weekly GLP-1 receptor agonist albiglutide (HARMONY 2): 52 week primary endpoint results from a randomised, placebo-controlled trial in patients with type 2 diabetes mellitus inadequately controlled with

diet and exercise. Diabetologia. 2016;59(2):266-74. Epub 2015/11/19. doi: 10.1007/s00125-015-3795-1. PubMed PMID: 26577795; PubMed Central PMCID: PMCPMC4705137.
26. Vanderheiden A, Harrison L, Warshauer J, Li X, Adams-Huet B, Lingvay I. Effect of Adding Liraglutide vs Placebo to a High-Dose lnsulin Regimen in Patients With Type 2 Diabetes: A Randomized Clinical Trial. JAMA Internal Medicine. 2016;176(7):939-47. doi: 10.1001/jamainternmed.2016.1540 %J JAMA Internal Medicine.
27. Ahmann A, Rodbard HW, Rosenstock J, Lahtela JT, de Loredo L, Tornøe K, et al. Efficacy and safety of liraglutide versus placebo added to basal insulin analogues (with or without metformin) in patients with type 2 diabetes: a randomized, placebo-controlled trial. Diabetes, obesity & metabolism. 2015;17(11):1056-64. Epub 2015/07/17. doi: 10.1111/dom.12539. PubMed PMID: 26179619; PubMed Central PMCID: PMCPMC5054929.
28. Davies MJ, Bergenstal R, Bode B, Kushner RF, Lewin A, Skjøth TV, et al. Efficacy of Liraglutide for Weight Loss Among Patients With Type 2 Diabetes: The SCALE Diabetes Randomized Clinical Trial. Jama. 2015;314(7):687-99. Epub 2015/08/19. doi: 10.1001/jama.2015.9676. PubMed PMID: 26284720.
29. Retnakaran R, Kramer CK, Choi H, Swaminathan B, Zinman B. Liraglutide and the preservation of pancreatic β-cell function in early type 2 diabetes: the LIBRA trial. Diabetes care. 2014;37(12):3270-8. Epub 2014/09/25. doi: 10.2337/dc14-0893. PubMed PMID: 25249651.
30. Kim SH, Abbasi F, Lamendola C, Liu A, Ariel D, Schaaf P, et al. Benefits of liraglutide treatment in overweight and obese older individuals with prediabetes. Diabetes care. 2013;36(10):3276-82. Epub 2013/07/10. doi: 10.2337/dc13-0354. PubMed PMID: 23835684; PubMed Central PMCID: PMCPMC3781545.
31. Wadden TA, Hollander P, Klein S, Niswender K, Woo V, Hale PM, et al. Weight maintenance and additional weight loss with liraglutide after low-calorie-diet-induced weight loss: the SCALE Maintenance randomized study. International journal of obesity (2005). 2013;37(11):1443-51. Epub 2013/07/03. doi: 10.1038/ijo.2013.120. PubMed PMID: 23812094.
