# Supplementary material for: Effect of glucagon-like peptide-1 receptor agonists on glycemic control, and weight reduction in adults: A multivariate meta-analysis
Source: PLoS One. 2023 Jan 25;18(1):e0278685. doi: 10.1371/journal.pone.0278685 (PMC9876280; doi:10.1371/journal.pone.0278685)
Supplement: S3 Table — (PDF) [file pone.0278685.s005.pdf]

S3 Table. Summary of risk of bias assessment for included studies.

Study	Randomization process	Deviations from intended interventions	Missing outcome data	Measurement of the outcome	Selection of the reported result	Overall
Davies, 2021[1]	Low	Low	Low	Low	Low	Low
Gudbergsen, 2021[2]	Low	Low	Low	Low	Low	Low
Nealand, 2021[3]	Low	Low	Some concerns*	Low	Low	Some concerns
Wadden, 2021[4]	Low	Low	Low	Low	Low	Low
Wilding, 2021[5]	Low	Low	Low	Low	Low	Low
Garvey, 2020[6]	Low	Low	Low	Low	Low	Low
Ghanim, 2020[7]	Low	Low	Some concerns*	Low	Low	Some concerns
Aroda, 2019[8]	Low	Low	Low	Low	Low	Low
Gerstein, 2019[9]	Low	Low	Low	Low	Low	Low
Husain, 2019[10]	Low	Low	Low	Low	Low	Low
Mosenzonl, 2019[11]	Low	Low	Low	Low	Low	Low
van Eyk, 2019[12]	Low	Low	Low	Low	Low	Low
Zinman, 2019 (sc)[13]	Low	Low	Low	Low	Low	Low
Zinman, 2019 (oral)[14]	Low	Low	Low	Low	Low	Low
Frøssing, 2018[15]	Low	Low	Low	Low	Low	Low
Rodbard, 2018[16]	Low	Low	Low	Low	Low	Low
Ishøy, 2017[17]	Low	Low	Some concerns*	Low	Low	Some concerns
Mensberg, 2017[18]	Low	Low	Some concerns*	Low	Low	Some concerns

Sorli, 2017[19]	Low	Low	Low	Low	Low	Low
Blackman, 2016[20]	Low	Low	Low	Low	Low	Low
Davies, 2016[21]	Low	Low	Low	Low	Low	Low
Dejgaard, 2016[22]	Low	Low	Low	Low	Low	Low
Kuhadiya, 2016[23]	Low	Low	Low	Low	Low	Low
Marso, 2016[24]	Low	Low	Low	Low	Low	Low
Nauck, 2016[25]	Low	Low	Low	Low	Low	Low
Vanderheiden, 2016[26]	Low	Low	Low	Low	Low	Low
Ahmann, 2015[27]	Low	Low	Low	Low	Low	Low
Davies, 2015[28]	Low	Low	Low	Low	Low	Low
Retnakaran, 2014[29]	Low	Low	Low	Low	Low	Low
Kim, 2013[30]	Low	Low	Some concerns*	Low	Low	Some concerns
Wadden, 2013[31]	Low	Low	Low	Low	Low	Low

* Not intention-to-treat analysis

Reference

1. Davies M, Færch L, Jeppesen OK, Pakseresht A, Pedersen SD, Perreault L, et al. Semaglutide 2·4 mg once a week in adults with overweight or obesity, and type 2 diabetes (STEP 2): a randomised, double-blind, double-dummy, placebo-controlled, phase 3 trial. The Lancet. 2021;397(10278):971-84. doi: 10.1016/S0140-6736(21)00213-0.

2. Gudbergsen H, Overgaard A, Henriksen M, Wæhrens EE, Bliddal H, Christensen R, et al. Liraglutide after diet-induced weight loss for pain and weight control in knee osteoarthritis: a randomized controlled trial. The American journal of clinical nutrition. 2021;113(2):314-23. Epub 2021/01/21. doi: 10.1093/ajcn/nqaa328. PubMed PMID: 33471039.

3. Neeland IJ, Marso SP, Ayers CR, Lewis B, Oslica R, Francis W, et al. Effects of liraglutide on visceral and ectopic fat in adults with overweight and obesity at high cardiovascular risk: a randomised, double-blind, placebo-controlled, clinical trial. The lancet Diabetes & endocrinology. 2021;9(9):595-605. Epub 2021/08/07. doi: 10.1016/s2213-8587(21)00179-0. PubMed PMID: 34358471.

4. Wadden TA, Bailey TS, Billings LK, Davies M, Frias JP, Koroleva A, et al. Effect of Subcutaneous Semaglutide vs Placebo as an Adjunct to Intensive Behavioral Therapy on Body Weight in Adults With Overweight or Obesity: The STEP 3 Randomized Clinical Trial. Jama. 2021;325(14):1403-13. Epub 2021/02/25. doi: 10.1001/jama.2021.1831. PubMed PMID: 33625476; PubMed Central PMCID: PMCPMC7905697 Nordisk (from grant support to the University of Pennsylvania) and personal fees from Novo Nordisk for service on a scientific advisory board during the conduct of the study, as well as personal fees from WW International (formerly Weight Watchers) for service on a scientific advisory board outside the submitted work. Dr Bailey reports receiving grants, personal fees, and nonfinancial support (writing assistance) from Novo Nordisk during the conduct of the study. Dr Billings reports receiving personal fees from Novo Nordisk, Sanofi, and Eli Lilly outside the submitted work. Dr Davies is co-funded by the NIHR Leicester Biomedical Research Centre and reports receiving consulting fees from Novo Nordisk; advisory board member, speaker, and consulting fees from Sanofi-Aventis, Eli Lilly, Merck Sharp & Dohme, Boehringer Ingelheim, AstraZeneca, and Janssen; advisory board fees from Lexicon, Servier, and Gilead Sciences Ltd; speaker fees from Napp Pharmaceuticals; and grants from Novo Nordisk, Sanofi-Aventis, Lilly, Boehringer Ingelheim, AstraZeneca, and Janssen outside the submitted work. Dr Frias reports receiving research support grants from Novo Nordisk during the conduct of the study; grants and personal fees from Boehringer Ingelheim, Eli Lilly, Merck, Novo Nordisk, and Sanofi; and grants from Janssen and Pfizer outside the submitted work. Dr Koroleva

reports being an employee of Novo Nordisk A/S and holding shares in the company. Dr Lingvay reports receiving advisory board fees and consulting fees from AstraZeneca, consulting fees from Bayer HealthCare Pharmaceuticals, Eli Lilly and Company, Intarcia, Intercept Pharmaceuticals, Janssen Global Services, MannKind Corporation, Target Pharma, Valeritas, and Zealand Pharma; advisory board fees from Boehringer Ingelheim and Sanofi US Services; grant support, paid to UT Southwestern, from Merck; grant support, paid to her institution, from Mylan Pharmaceuticals and Pfizer; grant support, paid to UT Southwestern; and advisory board fees, consulting fees, and travel support from Novo Nordisk. Dr O'Neil reports receiving grants from Novo Nordisk during the conduct of the study; grants from Epitomee Medical, Eli Lilly, and WW International; and personal fees from Robard, Gedeon Richter, WebMD, and Novo Nordisk outside the submitted work. Dr Rubino reports receiving writing assistance from Novo Nordisk during the conduct of the study; serving as a clinical investigator for AstraZeneca, Boehringer Ingelheim, and Novo Nordisk; and reports owning Novo Nordisk shares of stock outside the submitted work. Dr Skovgaard reports spousal employment at Novo Nordisk. Dr Wallenstein reports receiving personal fees from Novo Nordisk during the conduct of the study and outside the submitted work. Dr Garvey reports receiving grants from Novo Nordisk; serving as site principal investigator for the clinical trial, which was sponsored by his university during the conduct of the study; receiving grants from Lexicon and Pfizer outside the submitted work; and serving as an ad hoc consultant on advisory committees for Jazz Pharmaceuticals, Boehringer Ingelheim, Novo Nordisk, and Pfizer. In each instance, he received no financial compensation, nor was there a financial relationship. No other disclosures were reported.

5. Wilding JPH, Batterham RL, Calanna S, Davies M, Van Gaal LF, Lingvay I, et al. Once-Weekly Semaglutide in Adults with Overweight or Obesity. 2021;384(11):989-1002. doi: 10.1056/NEJMoa2032183. PubMed PMID: 33567185.

6. Garvey WT, Birkenfeld AL, Dicker D, Mingrone G, Pedersen SD, Satylganova A, et al. Efficacy and Safety of Liraglutide 3.0 mg in Individuals With Overweight or Obesity and Type 2 Diabetes Treated With Basal Insulin: The SCALE Insulin Randomized Controlled Trial. Diabetes care. 2020;43(5):1085-93. Epub 2020/03/07. doi: 10.2337/dc19-1745. PubMed PMID: 32139381; PubMed Central PMCID: PMCPMC7171937.

7. Ghanim H, Batra M, Green K, Abuaysheh S, Hejna J, Makdissi A, et al. Liraglutide treatment in overweight and obese patients with type 1 diabetes: A 26-week randomized controlled trial; mechanisms of weight loss. Diabetes, obesity & metabolism. 2020;22(10):1742-52. Epub 2020/05/20. doi: 10.1111/dom.14090. PubMed PMID: 32424935.

8. Aroda VR, Rosenstock J, Terauchi Y, Altuntas Y, Lalic NM, Morales Villegas EC, et al. PIONEER 1: Randomized Clinical Trial of the Efficacy and Safety of Oral Semaglutide Monotherapy in Comparison With Placebo in Patients With Type 2 Diabetes. Diabetes care. 2019;42(9):1724-32. Epub 2019/06/13. doi: 10.2337/dc19-0749. PubMed PMID: 31186300.
9. Gerstein HC, Colhoun HM, Dagenais GR, Diaz R, Lakshmanan M, Pais P, et al. Dulaglutide and cardiovascular outcomes in type 2 diabetes (REWIND): a double-blind, randomised placebo-controlled trial. Lancet (London, England). 2019;394(10193):121-30. Epub 2019/06/14. doi: 10.1016/s0140-6736(19)31149-3. PubMed PMID: 31189511.
10. Husain M, Birkenfeld AL, Donsmark M, Dungan K, Eliaschewitz FG, Franco DR, et al. Oral Semaglutide and Cardiovascular Outcomes in Patients with Type 2 Diabetes. The New England journal of medicine. 2019;381(9):841-51. Epub 2019/06/12. doi: 10.1056/NEJMoa1901118. PubMed PMID: 31185157.
11. Mosenzon O, Blicher TM, Rosenlund S, Eriksson JW, Heller S, Hels OH, et al. Efficacy and safety of oral semaglutide in patients with type 2 diabetes and moderate renal impairment (PIONEER 5): a placebo-controlled, randomised, phase 3a trial. The lancet Diabetes & endocrinology. 2019;7(7):515-27. Epub 2019/06/14. doi: 10.1016/s2213-8587(19)30192-5. PubMed PMID: 31189517.
12. van Eyk HJ, Paiman EHM, Bizino MB, de Heer P, Geelhoed-Duijvestijn PH, Kharagjitsingh AV, et al. A double-blind, placebo-controlled, randomised trial to assess the effect of liraglutide on ectopic fat accumulation in South Asian type 2 diabetes patients. Cardiovascular diabetology. 2019;18(1):87. Epub 2019/07/11. doi: 10.1186/s12933-019-0890-5. PubMed PMID: 31288820; PubMed Central PMCID: PMCPMC6615254.
13. Zinman B, Bhosekar V, Busch R, Holst I, Ludvik B, Thielke D, et al. Semaglutide once weekly as add-on to SGLT-2 inhibitor therapy in type 2 diabetes (SUSTAIN 9): a randomised, placebo-controlled trial. The lancet Diabetes & endocrinology. 2019;7(5):356-67. Epub 2019/03/06. doi: 10.1016/s2213-8587(19)30066-x. PubMed PMID: 30833170.
14. Zinman B, Aroda VR, Buse JB, Cariou B, Harris SB, Hoff ST, et al. Efficacy, Safety, and Tolerability of Oral Semaglutide Versus Placebo Added to Insulin With or Without Metformin in Patients With Type 2 Diabetes: The PIONEER 8 Trial. Diabetes care. 2019;42(12):2262-71. Epub 2019/09/19. doi: 10.2337/dc19-0898. PubMed PMID: 31530667; PubMed Central PMCID: PMCPMC7364672.
15. Frøssing S, Nylander M, Chabanova E, Frystyk J, Holst JJ, Kistorp C, et al. Effect of liraglutide on ectopic fat in polycystic ovary syndrome: A randomized clinical trial.

Diabetes, obesity & metabolism. 2018;20(1):215-8. Epub 2017/07/07. doi: 10.1111/dom.13053. PubMed PMID: 28681988.
16. Rodbard HW, Lingvay I, Reed J, de la Rosa R, Rose L, Sugimoto D, et al. Semaglutide Added to Basal Insulin in Type 2 Diabetes (SUSTAIN 5): A Randomized, Controlled Trial. The Journal of clinical endocrinology and metabolism. 2018;103(6):2291-301. Epub 2018/04/25. doi: 10.1210/jc.2018-00070. PubMed PMID: 29688502; PubMed Central PMCID: PMCPMC5991220.
17. Ishøy PL, Knop FK, Broberg BV, Bak N, Andersen UB, Jørgensen NR, et al. Effect of GLP-1 receptor agonist treatment on body weight in obese antipsychotic-treated patients with schizophrenia: a randomized, placebo-controlled trial. Diabetes, Obesity and Metabolism. 2017;19(2):162-71. doi: 10.1111/dom.12795. PubMed Central PMCID: PMCAstra Zeneca(Sweden).
18. Mensberg P, Nyby S, Jørgensen PG, Storgaard H, Jensen MT, Sivertsen J, et al. Near-normalization of glycaemic control with glucagon-like peptide-1 receptor agonist treatment combined with exercise in patients with type 2 diabetes. Diabetes, Obesity and Metabolism. 2017;19(2):172-80. doi: 10.1111/dom.12797. PubMed Central PMCID: PMCNovo Nordisk.
19. Sorli C, Harashima SI, Tsoukas GM, Unger J, Karsbøl JD, Hansen T, et al. Efficacy and safety of once-weekly semaglutide monotherapy versus placebo in patients with type 2 diabetes (SUSTAIN 1): a double-blind, randomised, placebo-controlled, parallel-group, multinational, multicentre phase 3a trial. The lancet Diabetes & endocrinology. 2017;5(4):251-60. Epub 2017/01/24. doi: 10.1016/s2213-8587(17)30013-x. PubMed PMID: 28110911.
20. Blackman A, Foster GD, Zammit G, Rosenberg R, Aronne L, Wadden T, et al. Effect of liraglutide 3.0 mg in individuals with obesity and moderate or severe obstructive sleep apnea: the SCALE Sleep Apnea randomized clinical trial. International journal of obesity (2005). 2016;40(8):1310-9. Epub 2016/03/24. doi: 10.1038/ijo.2016.52. PubMed PMID: 27005405; PubMed Central PMCID: PMCPMC4973216 Valeant Canada, Paladin Labs Inc. Dr Foster: at the time of the study was a scientific advisory board participant for Con Agra Foods, United Health Group and Tate and Lyle, and a consultant to Eisai and Novo Nordisk; currently employed by Weight Watchers International. Dr Zammit: consultant for Acorda, Actelion, Alexza, Arena, Aventis, Biovail, Boehringer-Ingelheim, Cephalon, Elan, Eli Lilly, Evotec, Forest, Glaxo Smith Kline, Jazz, King Pharmaceuticals, Ligand, McNeil, Merck, Neurocrine Biosciences, Organon, Pfizer, Purdue, Renovis, Sanofi-Aventis, Select Comfort, Sepracor, Shire, Somnus, Takeda Pharmaceuticals, Vela, Wyeth; grants/research support from Abbott, Abbvie, Actelion, Ancile, Apnex, Arena, Astra-Zeneca, Aventis, Banyu, Biomarin, BMS, Catalyst, Cephalon Inc., CHDI, Elan, Epix,

Eisai, Elminda, Evotec, Forest, Galderma, Genentech, Gilead, Glaxo Smith Kline, Gilead, H. Lundbeck A/S, Janssen, Johnson & Johnson, King, Merck and Co., National Institute of Health (NIH), Neurim, Neurocrine Biosciences, Naurex, Neurim, Neurogen, Novo Nordisk, Organon, Orphan Medical, Otsuka, Pfizer, Predix, Respironics, Saladax, Sanofi-Aventis, Sanofi-Synthelabo, Schering-Plough, Sepracor, Sunovion, Shire, Somaxon, Takeda Pharmaceuticals North America, Targacept, Teva, Thymon, Transcept, UCB Pharma, Ultragenyx, Predix, Vanda, Wyeth-Ayerst Research; honoraria received from Neurocrine Biosciences, King Pharmaceuticals, McNeil, Sanofi-Aventis, Sanofi-Synthelabo, Sepracor, Takeda Pharmaceuticals, Vela Pharmaceuticals, Wyeth-Ayerst Research; ownership interest in Clinilabs, Inc., Clinilabs IPA, Inc., Home Sleep and Respiratory Care, Nationwide Sleep Testing. Dr Rosenberg: received research grant support from Merck, Novo Nordisk, Pfizer, Teva, Jazz Pharmaceuticals, Sunovion Pharmaceuticals and Philips-Respironics. Dr Aronne: advisory board member for Myos Corporation; consultant, speaker, advisor, or received research support from Aspire Bariatrics Inc., Eisai, Ethicon Endo-Surgery Inc., GlaxoSmithKline Consumer Healthcare LP, GI Dynamics, Novo Nordisk, Pfizer, Takeda Pharmaceuticals, Vivus Inc., Zafgen Inc.; ownership interest in Myos Corporation and BMIQ. Dr Wadden: advisory board participant for Orexigen Pharmaceuticals, Inc., Novo Nordisk, Nutrisystem, and Shire Pharmaceuticals, with research support (to the University of Pennsylvania) from the first three companies. Dr Claudius: employee of Novo Nordisk and owns company stock. Dr Jensen: employee of Novo Nordisk and owns company stock. Dr Mignot: advisory board member, consultant for and research support from Jazz Pharmaceuticals; consultant for and research grant from Novo Nordisk; consultant for Reset Pharmaceuticals and Merck; expert witness for FTC.

21. Davies MJ, Bain SC, Atkin SL, Rossing P, Scott D, Shamkhalova MS, et al. Efficacy and Safety of Liraglutide Versus Placebo as Add-on to Glucose-Lowering Therapy in Patients With Type 2 Diabetes and Moderate Renal Impairment (LIRA-RENAL): A Randomized Clinical Trial. Diabetes care. 2016;39(2):222-30. Epub 2015/12/19. doi: 10.2337/dc14-2883. PubMed PMID: 26681713.

22. Dejgaard TF, Frandsen CS, Hansen TS, Almdal T, Urhammer S, Pedersen-Bjergaard U, et al. Efficacy and safety of liraglutide for overweight adult patients with type 1 diabetes and insufficient glycaemic control (Lira-1): A randomised, double-blind, placebo-controlled trial. The Lancet Diabetes and Endocrinology. 2016;4(3):221-32. doi: 10.1016/S2213-8587(15)00436-2. PubMed Central PMCID: PMCNovo Nordisk(Denmark).

23. Kuhadiya ND, Dhindsa S, Ghanim H, Mehta A, Makdissi A, Batra M, et al. Addition of Liraglutide to Insulin in Patients With Type 1 Diabetes: A Randomized

Placebo-Controlled Clinical Trial of 12 Weeks. Diabetes care. 2016;39(6):1027-35. Epub 2016/05/22. doi: 10.2337/dc15-1136. PubMed PMID: 27208343; PubMed Central PMCID: PMCPMC5864130.
24. Marso SP, Bain SC, Consoli A, Eliaschewitz FG, Jódar E, Leiter LA, et al. Semaglutide and Cardiovascular Outcomes in Patients with Type 2 Diabetes. 2016;375(19):1834-44. doi: 10.1056/NEJMoa1607141. PubMed PMID: 27633186.
25. Nauck MA, Stewart MW, Perkins C, Jones-Leone A, Yang F, Perry C, et al. Efficacy and safety of once-weekly GLP-1 receptor agonist albiglutide (HARMONY 2): 52 week primary endpoint results from a randomised, placebo-controlled trial in patients with type 2 diabetes mellitus inadequately controlled with diet and exercise. Diabetologia. 2016;59(2):266-74. Epub 2015/11/19. doi: 10.1007/s00125-015-3795-1. PubMed PMID: 26577795; PubMed Central PMCID: PMCPMC4705137.
26. Vanderheiden A, Harrison L, Warshauer J, Li X, Adams-Huet B, Lingvay I. Effect of Adding Liraglutide vs Placebo to a High-Dose lnsulin Regimen in Patients With Type 2 Diabetes: A Randomized Clinical Trial. JAMA Internal Medicine. 2016;176(7):939-47. doi: 10.1001/jamainternmed.2016.1540 %J JAMA Internal Medicine.
27. Ahmann A, Rodbard HW, Rosenstock J, Lahtela JT, de Loredo L, Tornøe K, et al. Efficacy and safety of liraglutide versus placebo added to basal insulin analogues (with or without metformin) in patients with type 2 diabetes: a randomized, placebo-controlled trial. Diabetes, obesity & metabolism. 2015;17(11):1056-64. Epub 2015/07/17. doi: 10.1111/dom.12539. PubMed PMID: 26179619; PubMed Central PMCID: PMCPMC5054929.
28. Davies MJ, Bergenstal R, Bode B, Kushner RF, Lewin A, Skjøth TV, et al. Efficacy of Liraglutide for Weight Loss Among Patients With Type 2 Diabetes: The SCALE Diabetes Randomized Clinical Trial. Jama. 2015;314(7):687-99. Epub 2015/08/19. doi: 10.1001/jama.2015.9676. PubMed PMID: 26284720.
29. Retnakaran R, Kramer CK, Choi H, Swaminathan B, Zinman B. Liraglutide and the preservation of pancreatic β-cell function in early type 2 diabetes: the LIBRA trial. Diabetes care. 2014;37(12):3270-8. Epub 2014/09/25. doi: 10.2337/dc14-0893. PubMed PMID: 25249651.
30. Kim SH, Abbasi F, Lamendola C, Liu A, Ariel D, Schaaf P, et al. Benefits of liraglutide treatment in overweight and obese older individuals with prediabetes. Diabetes care. 2013;36(10):3276-82. Epub 2013/07/10. doi: 10.2337/dc13-0354. PubMed PMID: 23835684; PubMed Central PMCID: PMCPMC3781545.
31. Wadden TA, Hollander P, Klein S, Niswender K, Woo V, Hale PM, et al. Weight maintenance and additional weight loss with liraglutide after low-calorie-diet-induced weight loss: the SCALE Maintenance randomized study. International journal

of obesity (2005). 2013;37(11):1443-51. Epub 2013/07/03. doi: 10.1038/ijo.2013.120. PubMed PMID: 23812094.
