# Supplementary material for: Effect of glucagon-like peptide-1 receptor agonists on glycemic control, and weight reduction in adults: A multivariate meta-analysis
Source: PLoS One. 2023 Jan 25;18(1):e0278685. doi: 10.1371/journal.pone.0278685 (PMC9876280; doi:10.1371/journal.pone.0278685)
Supplement: S4 Table — (PDF) [file pone.0278685.s006.pdf]

S4 Table. Sensitivity analysis of the pooled results for glycated hemoglobin level and weight reduction on comparison of glucagon-like peptide-1 receptor agonist and placebo by using the structural equation modeling multivariate meta-analysis according to participant characteristics.

	**All participants**			**Patients with diabetes**		
**Setting r = 0.1**	Estimates (95% CI)	p	I^2	Estimates (95% CI)	p	I^2
Glycated hemoglobin (%)	-0.85 (-1.03, -0.66)	< 0.001	99 %	-0.96 (0.09, -1.14)	< 0.001	96 %
Body weight (kg)	-4.03 (-5.10, -2.95)	< 0.001	99 %	-3.23 (0.32, -3.86)	< 0.001	95 %
tao of glycated hemoglobin	0.18 (0.07, 0.30)	0.002		0.13 (0.05, 0.03)	0.008	
tao of body weight	7.36 (3.10, 11.62)	0.001		1.77 (0.62, 0.56)	0.004	
Covariance	-0.48 (-1.02, 0.06)	0.081		0.16 (0.14, -0.12)	0.256	
Standardized correlation coefficient	-0.41			0.33		
**Setting r = 0.3**	All participants			Patients with diabetes		
Glycated hemoglobin (%)	-0.85 (-1.03, -0.66)	< 0.001	99 %	-0.96 (0.09, -1.14)	< 0.001	96 %
Body weight (kg)	-4.03 (-5.11, -2.95)	< 0.001	99 %	-3.23 (0.32, -3.87)	< 0.001	96 %
tao of glycated hemoglobin	0.18 (0.07, 0.30)	0.002		0.13 (0.05, 0.03)	0.008	
tao of body weight	7.37 (3.10, 11.64)	0.001		1.77 (0.62, 0.56)	0.004	
Covariance	-0.49 (-1.02, 0.05)	0.076		0.15 (0.14, -0.13)	0.308	
Standardized correlation coefficient	-0.42			0.30		
**Restricted to studies with low risk of bias**	All participants			Patients with diabetes		
Glycated hemoglobin (%)	-0.83 (-1.01, 0.09)	< 0.001	99 %	-0.96 (0.09, -1.14)	< 0.001	96 %
Body weight (kg)	-4.32(-5.46, 0.58)	< 0.001	99 %	-3.25 (0.35, -3.93)	< 0.001	96 %

tao of glycated hemoglobin	0.18 (0.06, 0.07)	0.002	0.13 (0.05, 0.03)	0.008
tao of body weight	7.26 (2.79, 2.28)	0.001	1.87 (0.68, 0.54)	0.006
Covariance	-0.48 (-1.02, 0.27)	0.081	0.16 (0.15, -0.13)	0.287
Standardized correlation coefficient	-0.42		0.32	

CI, confidence interval; *r,* the correlation coefficient between glycated hemoglobin and body weight changes within the study level; tao, the variance of effect measure. Further sensitivity analyses of participants without diabetes were not performed due to the limited included articles.
