# Supplementary figures and images for: Effect of glucagon-like peptide-1 receptor agonists on glycemic control, and weight reduction in adults: A multivariate meta-analysis
Source: PLoS One. 2023 Jan 25;18(1):e0278685. doi: 10.1371/journal.pone.0278685 (PMC9876280; doi:10.1371/journal.pone.0278685)

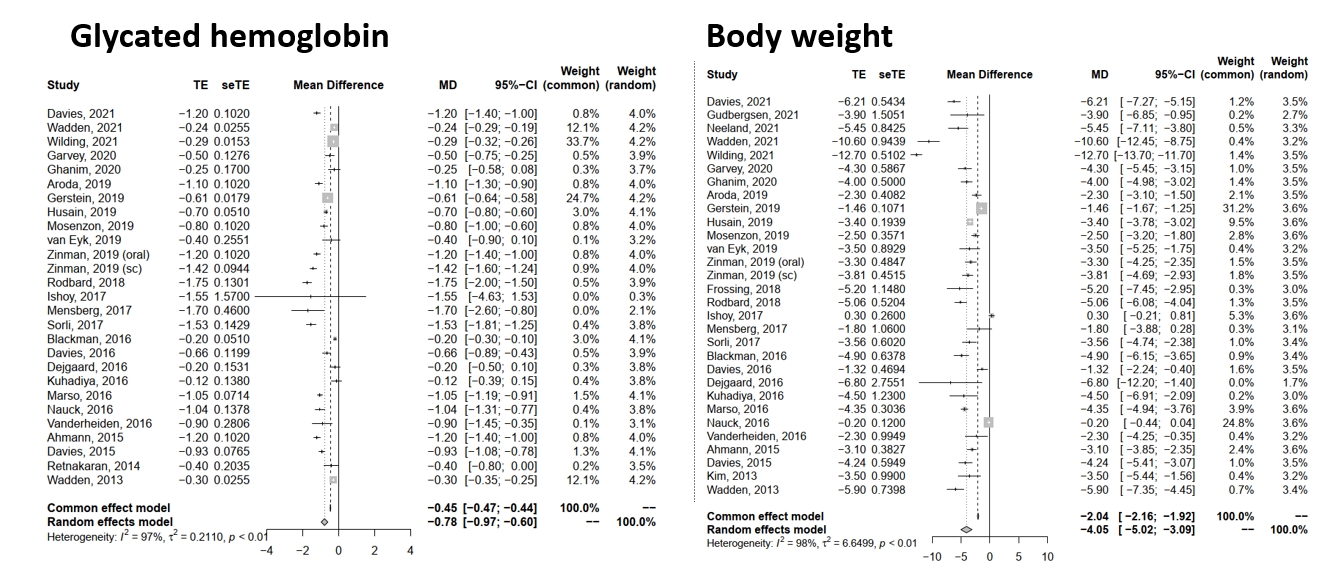

Supplement: S1 Fig — CI, confidence interval; MD, mean difference; SE, standard error; TE, treatment effect. (JPG) [file pone.0278685.s007.jpg]

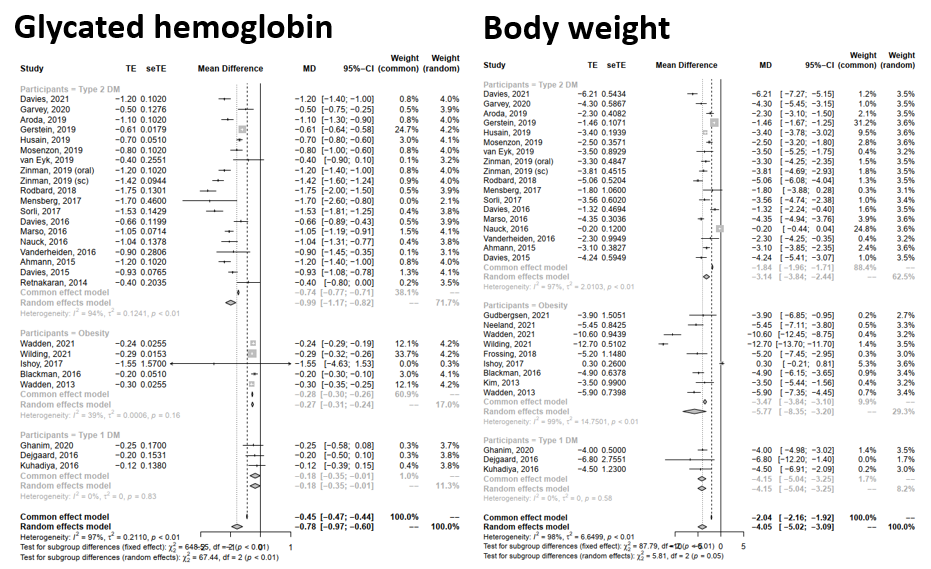

Supplement: S2 Fig — CI, confidence interval; DM, diabetes mellitus; MD, mean difference; SE, standard error; TE, treatment effect. (TIF) [file pone.0278685.s008.tif]

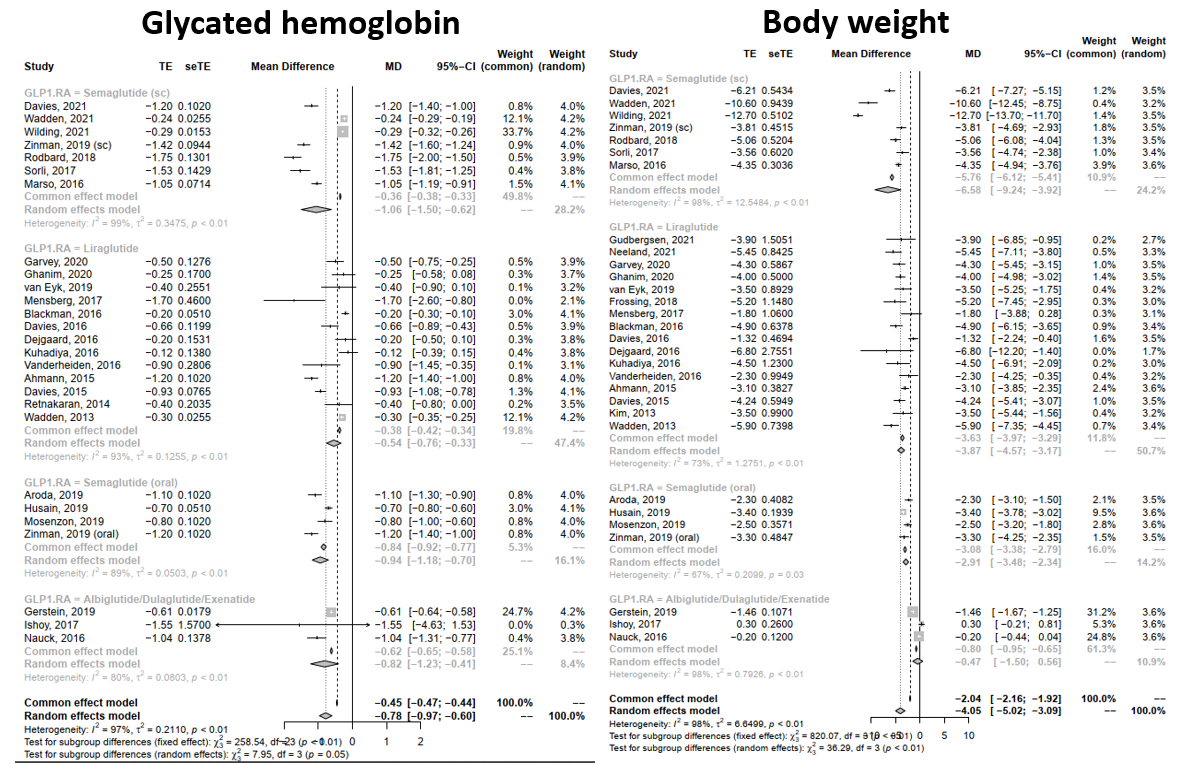

Supplement: S3 Fig — CI, confidence interval; GLP1 RA, Glucagon-like peptide-1 receptor agonist; MD, mean difference; sc, subcutaneous; SE, standard error; TE, treatment effect. GLP1 = Others referred to Dulaglutide, once-weekly Exenatide and Albiglutide. (TIF) [file pone.0278685.s009.tif]

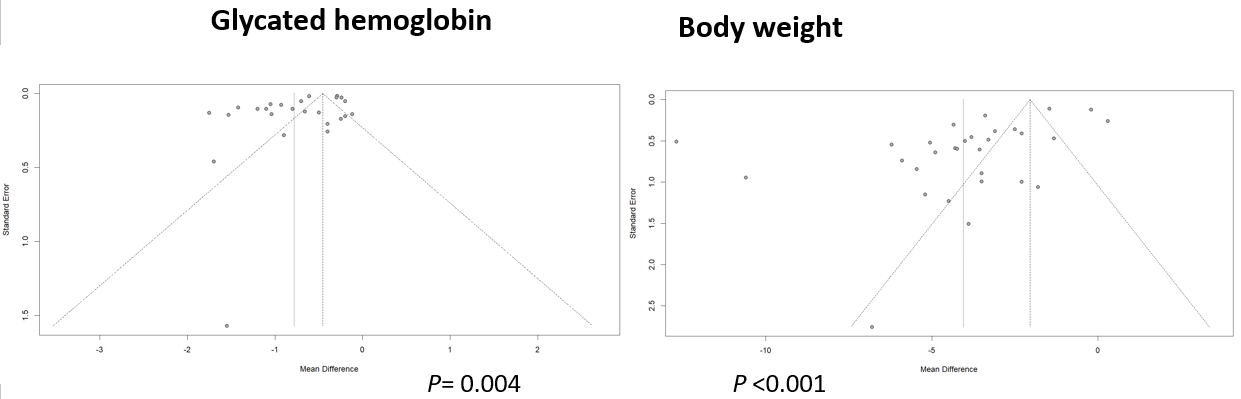

Supplement: S4 Fig — (TIF) [file pone.0278685.s010.tif]

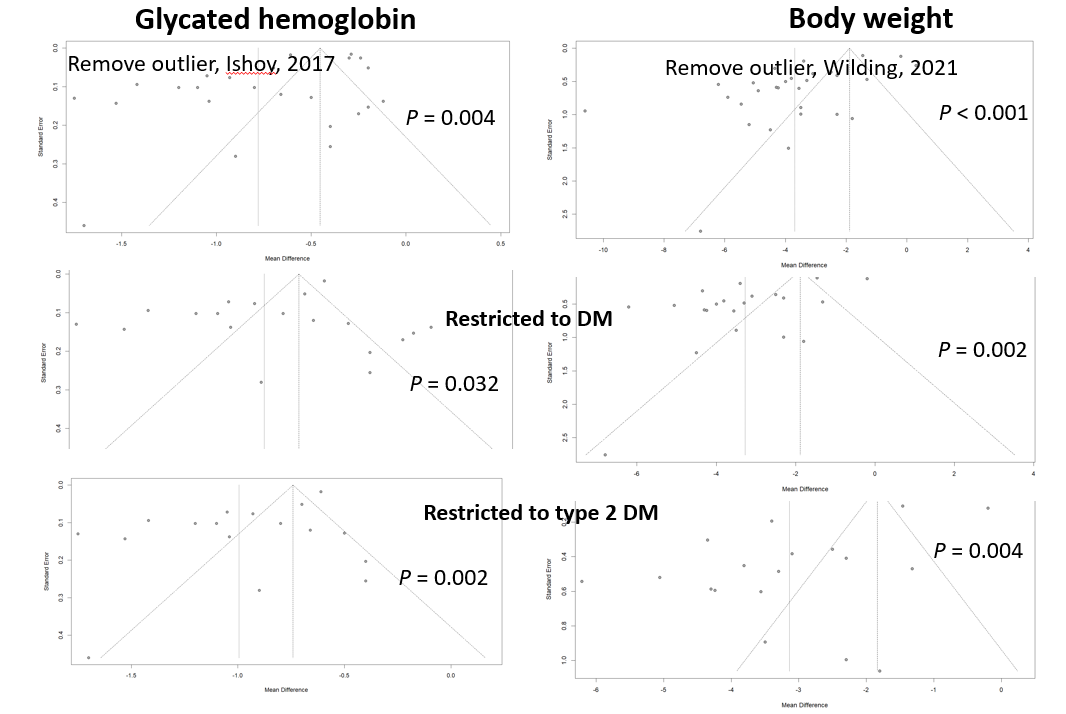

Supplement: S5 Fig — DM, diabetes mellitus; HbA1c, glycated hemoglobin. (TIF) [file pone.0278685.s011.tif]

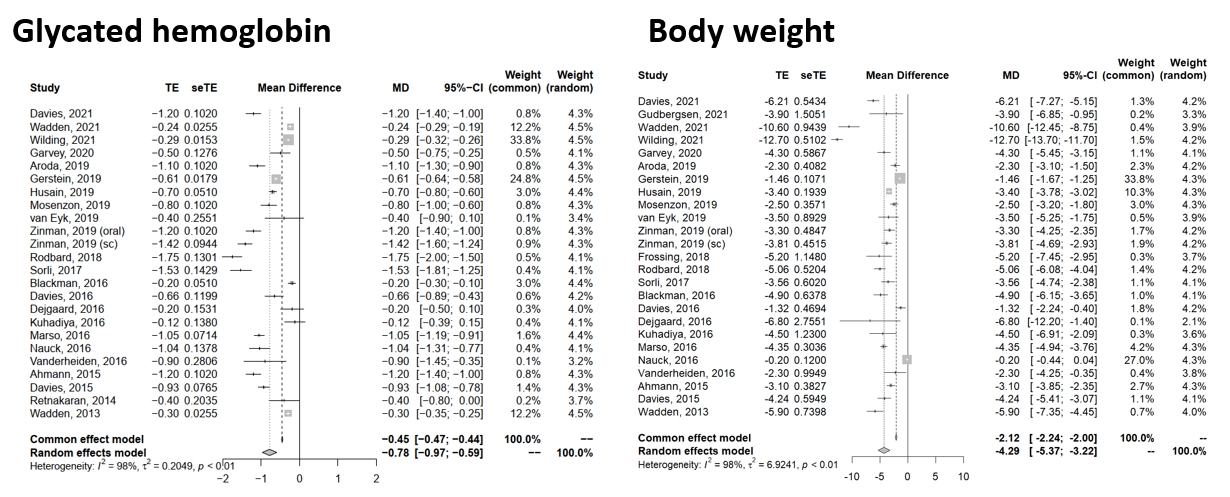

Supplement: S6 Fig — CI, confidence interval; MD, mean difference; SE, standard error; TE, treatment effect. (JPG) [file pone.0278685.s012.jpg]
