# Supplementary material for: Effect of glucagon-like peptide-1 receptor agonists on glycemic control, and weight reduction in adults: A multivariate meta-analysis
Source: PLoS One. 2023 Jan 25;18(1):e0278685. doi: 10.1371/journal.pone.0278685 (PMC9876280; doi:10.1371/journal.pone.0278685)
Supplement: Attachment — Submitted filename: REVIEWERS COMMENTS- PONE-D-22-25301.docx [file pone.0278685.s013.docx]

**RE: PONE-D-22-25301**

1. Title: Ambiguous- the authors need to modify the title. We suggest “Effect of glucagon-like peptide-1 receptor agonists on glycemic control, and weight reduction in adults: a multivariate meta-analysis”
2. Abstract
a) Aims: Requires modification to be in consonance with the new title
b) Methods: First sentence- The authors should state the exact time of commencement of the study and not just state “from study inception”.
c) Conclusion: The last sentence should be moved to the results section. However, the implication(s) of the result contained in the sentence can be included in the conclusion section as done in the conclusion section of the manuscript.
3. Introduction: Well written
4. Materials and Methods:
a) Line 61: There is the need for the authors to define the word “inception” and state categorically the date the study commenced OR did the authors mean since the inception of the databases? If yes, this should be expressly stated.
b) Lines 83 – 85: The last sentence in the methodology section should be moved to appropriate place as it described specific roles of some authors.
5. Data analysis: This section is too long. I would suggest that the authors revise lines 88 – 99 or move it to the supplementary section..
6. Results:
a) Table 1: The authors should unbold the results in bold format. The contents of the Table are already explained in lines 155 – 159 and the p-values are self-explanatory.
b) There is the need to ensure uniform decimal points for the p-values.
c) The authors should remove the sentence “bold indicates statistical significance” from the legend since the results will be unbolded.
d) Going through row 3 in Table 1, Age vs Body weight has p-value of 0.072 which is not significant but was bolded.
7. Discussion:
a) Line 263- Long-acting GLP-1 Ras “showed” and not “show”.
8. Conclusion: Sentence 2- Replace “could” with “might”

Other Comments

1. Inclusion of withdrawn, terminated and unknown status studies as stated in lines 278 – 280 is a source of concern as they could obscure the true findings and conclusion of this study. Is it possible for the authors to reanalyze the retrieved data without the aforementioned studies and find out if a similar outcome will still emerge?
2. Did the authors exclude studies that included type 2 diabetics on insulin in the multivariate analysis as this could affect both the glycemic control and body weight?
3. The observed negative correlation between glycemic control and weight reduction in the univariate analysis is not unexpected because of the diverse population of the participants, different types of GLP-1 RAs and the publication bias.

Suggestion

This manuscript might benefit from a Biostatistician input(s) as I have limited knowledge on the statistical tools/methods used in the study.
