# Supplementary material for: Effect of glucagon-like peptide-1 receptor agonists on glycemic control, and weight reduction in adults: A multivariate meta-analysis
Source: PLoS One. 2023 Jan 25;18(1):e0278685. doi: 10.1371/journal.pone.0278685 (PMC9876280; doi:10.1371/journal.pone.0278685)
Supplement: Attachment — Submitted filename: Response to Reviewers.docx [file pone.0278685.s014.docx]

**Response to reviewers**:

**PONE-D-22-25301**

**Association between glycemic control and weight reduction of glucagon-like peptide-1 receptor agonists: a multivariate meta-analysis**

When submitting your revision, we need you to address these additional requirements.

1. Please ensure that your manuscript meets PLOS ONE's style requirements, including those for file naming. The PLOS ONE style templates can be found at

https://journals.plos.org/plosone/s/file?id=wjVg/PLOSOne_formatting_sample_main_body.pdf and

https://journals.plos.org/plosone/s/file?id=ba62/PLOSOne_formatting_sample_title_authors_affiliations.pdf

**Response:** Thanks for your advice. We had changed the file names of the supplements.

2. Thank you for stating the following financial disclosure:

"The funders had no role in study design, data collection and analysis, decision to publish, or preparation of the manuscript."

At this time, please address the following queries:

a) Please clarify the sources of funding (financial or material support) for your study. List the grants or organizations that supported your study, including funding received from your institution.

b) State what role the funders took in the study. If the funders had no role in your study, please state: “The funders had no role in study design, data collection and analysis, decision to publish, or preparation of the manuscript.”

c) If any authors received a salary from any of your funders, please state which authors and which funders.

d) If you did not receive any funding for this study, please state: “The authors received no specific funding for this work.”

Please include your amended statements within your cover letter; we will change the online submission form on your behalf.

**Response:** Thanks for your advice. We included our amended statements within the **cover letter** that “**The authors received no specific funding for this work**.”

3. We note that this manuscript is a systematic review or meta-analysis; our author guidelines therefore require that you use PRISMA guidance to help improve reporting quality of this type of study. Please upload copies of the completed PRISMA checklist as Supporting Information with a file name “PRISMA checklist” and it should be uploaded separately.

**Response:** Thanks for your advice. We upload copies of the completed PRISMA checklist as Supporting Information with a file name “**PRISMA checklist**” and it **was uploaded separately**.

Reviewers' comments:

Comments to the Author

1. Is the manuscript technically sound, and do the data support the conclusions?

The manuscript must describe a technically sound piece of scientific research with data that supports the conclusions. Experiments must have been conducted rigorously, with appropriate controls, replication, and sample sizes. The conclusions must be drawn appropriately based on the data presented.

Reviewer #1: Yes

Reviewer #2: Partly

Reviewer #3: Yes

2. Has the statistical analysis been performed appropriately and rigorously?

Reviewer #1: Yes

Reviewer #2: Yes

Reviewer #3: I Don't Know

3. Have the authors made all data underlying the findings in their manuscript fully available?

The PLOS Data policy requires authors to make all data underlying the findings described in their manuscript fully available without restriction, with rare exception (please refer to the Data Availability Statement in the manuscript PDF file). The data should be provided as part of the manuscript or its supporting information, or deposited to a public repository. For example, in addition to summary statistics, the data points behind means, medians and variance measures should be available. If there are restrictions on publicly sharing data—e.g. participant privacy or use of data from a third party—those must be specified.

Reviewer #1: Yes

Reviewer #2: No

Reviewer #3: Yes

4. Is the manuscript presented in an intelligible fashion and written in standard English?

PLOS ONE does not copyedit accepted manuscripts, so the language in submitted articles must be clear, correct, and unambiguous. Any typographical or grammatical errors should be corrected at revision, so please note any specific errors here.

Reviewer #1: Yes

Reviewer #2: Yes

Reviewer #3: Yes

5. Review Comments to the Author

Please use the space provided to explain your answers to the questions above. You may also include additional comments for the author, including concerns about dual publication, research ethics, or publication ethics. (Please upload your review as an attachment if it exceeds 20,000 characters)

**Reviewer #1:**

The manuscript is to evaluate effects of GLP1R agonists on HbA1c and body weight reductions in real world population by meta-analysis methodology. Authors found positive association between HbA1c and body weight reductions by GLP1R agonists treatment in a diabetes patient population. I have a couple of critiques authors should address.

1. HbA1c reduction by GLP1R agonists treatment is interacted with the baseline HbA1c. Therefore, restricting population to DM patients skews associations between body weight and HbA1c effects to make another bias. Authors should touch on the issue.

**Response:** Thanks for your advice. We revised the **discussion** to touch on the issue: However, marked weight loss in a non-diabetic patient in response to GLP-1 RA treatment did not indicate that clinicians could expect a corresponding glycemic improvement due to the results **were interacted with the underlying HbA1c level ~~genetic polymorphisms~~.**

2. Authors do not take adverse effects into account in the analyses. Nausea is well-known adverse effect for GLP1R agonists. Hence, the AE could reduce appetite to modify body weight in entire population. Is there any interaction between AE and body weight?

**Response:** Thanks for your advice. Considering GI side effect could reduce appetite to modify body weight in entire population, we performed the **interaction between AE and outcomes** and the results were shown in Table 1.

Revised **results**: Meta-regression (Table 1) showed that the **pooled HbA1c reduction** significantly interacted with participants’ baseline age (p = 0.032), …, **and gastrointestinal side effect (p = 0.002)** but did not interact with ….. ; a 1% increase in the proportion of female participants significantly increased the pooled HbA1c change by 0.015%; …. and **1% increase in gastrointestinal side effect significantly increase the pooled HbA1c level by 0.017%.**…………….

Meta-regression (**Table 1**) showed that the **pooled body weight reduction** significantly interacted with the proportion of female participants (*p* = 0.016),…and **gastrointestinal side effect (*p* = 0.025)**, and ... A 1% increase in the proportion of female participants significantly decreased the pooled body weight reduction by 0.065 kg; …..; **a 1% increase in gastrointestinal side effect significantly decreased the pooled body weight reduction by 0.059 kg.**

**Table 1 Univariate meta-regression of the effects of glucagon-like peptide-1 receptor agonists on glycated hemoglobin levels and weight reduction**

|  | Glycated hemoglobin | | | Body weight | | |
| --- | --- | --- | --- | --- | --- | --- |
|  | MD (95% CI) | *p* | R^2^(%) | MD (95% CI) | *p* | R^2^(%) |
| Age (years) | **-0.026 (-0.049, -0.002)** | **0.032** | **17.2** | 0.093 (-0.016, 0.202) | **0.091** | 6.0 |
| Proportion of women (%) | **0.015 (0.003, 0.027)** | **0.017** | **17.3** | **-0.065 (-0.117, -0.013)** | **0.016** | **18.0** |
| Baseline glycated hemoglobin (%) | **-0.262 (-0.473, -0.050)** | **0.018** | **25.1** | 1.130 (-0.132, 2.393) | 0.077 | 9.6 |
| Baseline body mass index (kg/m^2^) | 0.021 (-0.045, 0.087) | 0.514 | 0 | -0.217 (-0.553, 0.120) | 0.197 | 3.5 |
| Baseline body weight (kg) | 0.010 (-0.013, 0.033) | 0.374 | 1.8 | -0.045 (-0.157, 0.068) | 0.424 | 0 |
| Duration of diabetes (years) | -0.004 (-0.035, 0.027) | 0.788 | 0 | 0.105 (-0.060, 0.269) | 0.203 | 3.7 |
| Study duration (weeks) | 0.002 (-0.007, 0.011) | 0.660 | 0 | **-0.052 (-0.094, -0.010)** | **0.018** | **16.2** |
| Gastrointestinal side effect (%) | 0.017 (0.007, 0.027) | 0.002 | 35.1 | **-0.059 (-0.110, -0.008)** | **0.025** | **15.1** |
| Insulin use (%) | 0.000 (-0.005, 0.005) | 0.863 | 0 | **0.015 (-0.011, 0.040)** | **0.249** | **2.2** |

CI, confidence interval; MD, mean difference**;** R^2^ (%), percentage of heterogeneity explained; bold indicates statistical significance

3. Line 296-321; Authors discuss about genetic polymorphisms as the potential reasons for different HbA1c responses. I do not understand the logic here. What kind of genetic polymorphisms is authors suggesting? The cited paper does not explain any polymorphisms for the GLP1R agonist responses. Is it a bit too haste to discuss about genetic differences for the different HbA1c effects?

**Response:** Thanks for your advice. We **deleted** the sentences. The revised discussion: The mechanism underlying the variable effects of GLP-1 RAs on body weight is not well understood. GLP-1 RAs decreased appetite through direct effects on the hypothalamus, neuronal activation in brain areas, reduced caloric intake, and interference of effective compensatory mechanisms counteracting weight loss.[60, 61].[1, 2] **~~Genetic polymorphisms through signal transduction pathways from different hypothalamic effects in GLP-1 RA responders and non-responders[63] partially explained this difference.~~**

….

Lin 304: However, marked weight loss in a non-diabetic patient in response to GLP-1 RA treatment did not indicate that clinicians could expect a corresponding glycemic improvement due to the results **were interacted with the underlying HbA1c level ~~genetic polymorphisms~~.**

4. Line 216-221; Numbers here do not match to the table 2. Please double check.

**Response:** Thanks for your advice. We matched the numbers in the results with the table 2. The revised results:

**Results of the structural equation modeling multivariate meta-analysis**

Maximum likelihood estimation worked well in the analysis. **Table** **2** shows that the pooled HbA1c change induced by GLP1-RAs was -0.85% (95% CI [-1.03%, -0.66%], *I^2^* = 99%), and the pooled body weight change was -4.03 kg (95% CI [-5.11 kg, -2.95 kg], *I^2^* = 99%), which were similar to the results of the univariate meta-analysis. However, overall, the pooled between-study level correlation coefficient between HbA1c and body weight changes from baseline was -0.42, which was the opposite of the within-study level. To explore the negative correlation, we further restricted the multivariate analysis to participants with diabetes. The pooled HbA1c change by GLP1-RA was -0.96% (95% CI [-1.14%, -0.79%], *I^2^* = 96%), the pooled body weight change was -3.23 kg (95% CI [-3.86 kg, -2.59 kg], *I^2^* = 95%); the amount of between-study heterogeneity of body weight decreased from 7.36 to 1.77 and the 95% CI became narrower. The pooled correlation coefficient turned to a positive estimate of 0.32. And if we restricted the multivariate analysis to participants without diabetes of only five studies. The pooled HbA1c change by GLP1-RA was -0.27% (95% CI [-0.31%, -0.23%], *I^2^* = 13%), the pooled body weight change was -6.76 kg (95% CI [-10.81 kg, -2.72 kg], *I^2^* = 99%); the amount of between-study heterogeneity of body weight was much increased to 20.84 with a wide 95% CI due to the limited included articles. However, the pooled correlation coefficient was positive estimate of 0.81.

**Table 2 The pooled results for glycated hemoglobin level and weight reduction on comparison of glucagon-like peptide-1 receptor agonist and placebo by using the structural equation modeling multivariate meta-analysis according to participant characteristics**

|  | All participants | | | Patients with diabetes | | | Patients without diabetes | | |
| --- | --- | --- | --- | --- | --- | --- | --- | --- | --- |
| Setting *r* = 0.2 | Estimates (95% CI) | *p* | *I^2^* | Estimates (95% CI) | *p* | *I^2^* | Estimates (95% CI) | *p* | *I^2^* |
| Glycated hemoglobin (%) | -0.85 (-1.03, -0.66) | <0.001 | 99 % | -0.96 (-1.14, -0.79) | <0.001 | 96 % | -0.27 (-0.31, -0.23) | <0.001 | 13 % |
| Body weight (kg) | -4.03 (-5.11, -2.95) | <0.001 | 99 % | -3.23 (-3.86, -2.59) | <0.001 | 95 % | -6.76 (-10.81, -2.72) | 0.001 | 99 % |
| tao of glycated hemoglobin | 0.18 (0.07, 0.30) | <0.001 |  | 0.13 (0.03, 0.23) | 0.008 |  | 0 | 0.909 |  |
| tao of body weight | 7.36 (3.10, 11.63) | <0.001 |  | 1.77 (0.56, 2.99) | 0.004 |  | 20.84 (-5.32, 47.00) | 0.118 |  |
| Covariance | -0.48 (-1.02, 0.05) | <0.001 |  | 0.15 (-0.12, 0.43) | 0.281 |  | 0.04 (-0.14, 0.21) | 0.682 |  |
| Standardized correlation coefficient | -0.42 |  |  | 0.32 |  |  | 0.81 |  |  |

CI, confidence interval; *r,* correlation coefficient between glycated hemoglobin and body weight changes within the study level; tao, the variance of effect measure

5. Reference #57 is irrelevant.

**Response:** Thanks for your advice. We **deleted** the reference #57

Thus, meta-analyses published before 2015….., while more recent meta-analyses usually targeted semaglutide[3-5] or focused on emerging outcomes such as cardiovascular or kidney disease.[6, 7] In contrast, our study aimed to investigate the glycemic control and weight reduction caused by long-acting GLP-1 RAs. Previous studies showed high heterogeneity (*I^2^* = 80%-90%) even for findings related to the same GLP-1 RAs. [3-5] All the potential effect modifiers in our study showed an opposite direction of interaction between glycemic control and weight reduction. Previous meta-analyses have rarely reported this topic and yielded inconsistent results[3-5];

**~~Reference 57.~~**	~~Li J, He K, Ge J, Li C, Jing Z. Efficacy and safety of the glucagon-like peptide-1 receptor agonist oral semaglutide in patients with type 2 diabetes mellitus: A systematic review and meta-analysis. Diabetes research and clinical practice. 2021;172:108656. Epub 2021/01/13. doi: 10.1016/j.diabres.2021.108656. PubMed PMID: 33434602.~~

6. Table 1; Baseline BMI is not interacted with GLP1R agonists mediated body weight changes. How about to use baseline body weight?

**Response:** Thanks for your advice. We performed **meta-regression** with **baseline body weight** with the outcomes…

**Revised** results:

Meta-regression (**Table 1**) showed that the **pooled HbA1c reduction** significantly interacted with participants’ baseline age (*p* = 0.032), proportion of female participants (*p* = 0.017), the baseline HbA1c level (*p* = 0.018), and gastrointestinal side effect (*p* = 0.002) but **did not interact with baseline body weight or BMI level**, duration of diabetes, follow-up period or insulin use.

Meta-regression (**Table 1**) showed that the pooled **body weight** reduction significantly interacted with the proportion of female participants (*p* = 0.016), the follow-up period (*p* = 0.018) and gastrointestinal side effect (*p* = 0.025), and had a borderline interaction with participants’ baseline age (*p* = 0.091) and the participants’ baseline HbA1c level (*p* = 0.077)**, but did not interact with baseline BMI level or body weight,** insulin use, or the duration of diabetes.

**Table 1 Univariate meta-regression of the effects of glucagon-like peptide-1 receptor agonists on glycated hemoglobin levels and weight reduction**

|  | Glycated hemoglobin | | | Body weight | | |
| --- | --- | --- | --- | --- | --- | --- |
|  | MD (95% CI) | *p* | R^2^(%) | MD (95% CI) | *p* | R^2^(%) |
| Age (years) | **-0.026 (-0.049, -0.002)** | **0.032** | **17.2** | 0.093 (-0.016, 0.202) | **0.091** | 6.0 |
| Proportion of women (%) | **0.015 (0.003, 0.027)** | **0.017** | **17.3** | **-0.065 (-0.117, -0.013)** | **0.016** | **18.0** |
| Baseline glycated hemoglobin (%) | **-0.262 (-0.473, -0.050)** | **0.018** | **25.1** | 1.130 (-0.132, 2.393) | 0.077 | 9.6 |
| Baseline body mass index (kg/m^2^) | 0.021 (-0.045, 0.087) | 0.514 | 0 | -0.217 (-0.553, 0.120) | 0.197 | 3.5 |
| Baseline body weight (kg) | 0.010 (-0.013, 0.033) | 0.374 | 1.8 | -0.045 (-0.157, 0.068) | 0.424 | 0 |
| Duration of diabetes (years) | -0.004 (-0.035, 0.027) | 0.788 | 0 | 0.105 (-0.060, 0.269) | 0.203 | 3.7 |
| Study duration (weeks) | 0.002 (-0.007, 0.011) | 0.660 | 0 | **-0.052 (-0.094, -0.010)** | **0.018** | **16.2** |
| Gastrointestinal side effect (%) | 0.017 (0.007, 0.027) | 0.002 | 35.1 | **-0.059 (-0.110, -0.008)** | **0.025** | **15.1** |
| Insulin use (%) | 0.000 (-0.005, 0.005) | 0.863 | 0 | **0.015 (-0.011, 0.040)** | **0.249** | **2.2** |

CI, confidence interval; MD, mean difference**;** R^2^ (%), percentage of heterogeneity explained; bold indicates statistical significance

7. Table 2 and S5 Table; What is a definition of tao?

**Response:** Thanks for your advice. We revised the footnotes of the tables of table 2 and S5 table.

**tao, the variance of effect measure**

**Table 2 The pooled results for glycated hemoglobin level and weight reduction on comparison of glucagon-like peptide-1 receptor agonist and placebo by using the structural equation modeling multivariate meta-analysis according to participant characteristics**

|  | All participants | | | Patients with diabetes | | | Patients without diabetes | | |
| --- | --- | --- | --- | --- | --- | --- | --- | --- | --- |
| Setting *r* = 0.2 | Estimates (95% CI) | *p* | *I^2^* | Estimates (95% CI) | *p* | *I^2^* | Estimates (95% CI) | *p* | *I^2^* |
| Glycated hemoglobin (%) | -0.85 (-1.03, -0.66) | <0.001 | 99 % | -0.96 (-1.14, -0.79) | <0.001 | 96 % | -0.27 (-0.31, -0.23) | <0.001 | 13 % |
| Body weight (kg) | -4.03 (-5.11, -2.95) | <0.001 | 99 % | -3.23 (-3.86, -2.59) | <0.001 | 95 % | -6.76 (-10.81, -2.72) | 0.001 | 99 % |
| tao of glycated hemoglobin | 0.18 (0.07, 0.30) | <0.001 |  | 0.13 (0.03, 0.23) | 0.008 |  | 0 | 0.909 |  |
| tao of body weight | 7.36 (3.10, 11.63) | <0.001 |  | 1.77 (0.56, 2.99) | 0.004 |  | 20.84 (-5.32, 47.00) | 0.118 |  |
| Covariance | -0.48 (-1.02, 0.05) | <0.001 |  | 0.15 (-0.12, 0.43) | 0.281 |  | 0.04 (-0.14, 0.21) | 0.682 |  |
| Standardized correlation coefficient | -0.42 |  |  | 0.32 |  |  | 0.81 |  |  |

CI, confidence interval; *r,* correlation coefficient between glycated hemoglobin and body weight changes within the study level; **tao, the variance of effect measure**

**S4 Table. Sensitivity analysis of the pooled results for glycated hemoglobin level and weight reduction on comparison of glucagon-like peptide-1 receptor agonist and placebo by using the structural equation modeling multivariate meta-analysis according to participant characteristics.**

|  | **All participants** | | | | **Patients with diabetes** | | | | | |
| --- | --- | --- | --- | --- | --- | --- | --- | --- | --- | --- |
| **Setting *r* = 0.1** | Estimates (95% CI) | *p* | *I^2^* | Estimates (95% CI) | | | *p* | *I^2^* | |  |
| Glycated hemoglobin (%) | -0.85 (-1.03, -0.66) | < 0.001 | 99 % | -0.96 (0.09, -1.14) | | < 0.001 | | | 96 % |  |
| Body weight (kg) | -4.03 (-5.10, -2.95) | < 0.001 | 99 % | -3.23 (0.32, -3.86) | | < 0.001 | | | 95 % |  |
| tao of glycated hemoglobin | 0.18 (0.07, 0.30) | 0.002 |  | 0.13 (0.05, 0.03) | | 0.008 | | |  |  |
| tao of body weight | 7.36 (3.10, 11.62) | 0.001 |  | 1.77 (0.62, 0.56) | | 0.004 | | |  |  |
| Covariance | -0.48 (-1.02, 0.06) | 0.081 |  | 0.16 (0.14, -0.12) | | 0.256 | | |  |  |
| Standardized correlation coefficient | -0.41 |  |  | 0.33 | |  | | |  |  |
| **Setting *r* = 0.3** | All participants | | | | Patients with diabetes | | | | | |
| Glycated hemoglobin (%) | -0.85 (-1.03, -0.66) | < 0.001 | 99 % | -0.96 (0.09, -1.14) | | < 0.001 | | | 96 % |  |
| Body weight (kg) | -4.03 (-5.11, -2.95) | < 0.001 | 99 % | -3.23 (0.32, -3.87) | | < 0.001 | | | 96 % |  |
| tao of glycated hemoglobin | 0.18 (0.07, 0.30) | 0.002 |  | 0.13 (0.05, 0.03) | | 0.008 | | |  |  |
| tao of body weight | 7.37 (3.10, 11.64) | 0.001 |  | 1.77 (0.62, 0.56) | | 0.004 | | |  |  |
| Covariance | -0.49 (-1.02, 0.05) | 0.076 |  | 0.15 (0.14, -0.13) | | 0.308 | | |  |  |
| Standardized correlation coefficient | -0.42 |  |  | 0.30 | |  | | |  |  |
| **Restricted to** **studies with low risk of bias** | All participants | | | | Patients with diabetes | | | | | |
| Glycated hemoglobin (%) | -0.83 (-1.01, 0.09) | < 0.001 | 99 % | -0.96 (0.09, -1.14) | | < 0.001 | | | 96 % |  |
| Body weight (kg) | -4.32(-5.46, 0.58) | < 0.001 | 99 % | -3.25 (0.35, -3.93) | | < 0.001 | | | 96 % |  |
| tao of glycated hemoglobin | 0.18 (0.06, 0.07) | 0.002 |  | 0.13 (0.05, 0.03) | | 0.008 | | |  |  |
| tao of body weight | 7.26 (2.79, 2.28) | 0.001 |  | 1.87 (0.68, 0.54) | | 0.006 | | |  |  |
| Covariance | -0.48 (-1.02, 0.27) | 0.081 |  | 0.16 (0.15, -0.13) | | 0.287 | | |  |  |
| Standardized correlation coefficient | -0.42 |  |  | 0.32 | |  | | |  |  |

CI, confidence interval; *r,* the correlation coefficient between glycated hemoglobin and body weight changes within the study level; **tao, the variance of effect measure. Further sensitivity analyses of participants without diabetes was not performed due to the limited included article**

**Reviewer #2:**

With reference to the review of Manuscript number PONE-D-22-25301 “Association between glycemic control and weight reduction of glucagon-like peptide-1 receptor agonists: a multivariate meta-analysis”

Following views are there:

A useful study in a way that obese adults are prone to development of diabetes the study shows that glucagon-like peptide-1 receptor agonists improve glycaemia and decreases the body weight, both effects can protect obese adults from developing diabetes.

However many background con founders can effect the weight gain or loss therefore stratification is desired like patient with and without background Insulin treatment will have different effect on body weight reduction. Different duration of treatment can affect body weight differently. Kindly include non diabetic patients in Table 2 ,5. (All Participants, diabetic and non diabetic participants.)

**Response:** Thanks for your advice, we performed further analyses to all participants, patients with diabetes and **participants without diabetes.**

**Revised results: Table 2** shows that the pooled HbA1c change induced by GLP1-RAs was…. To explore the negative correlation, we further restricted the multivariate analysis to participants with diabetes. …. **There were only five studies focused on participants without diabetes. The pooled HbA1c change by GLP1-RA was -0.27% (95% CI [-0.31%, -0.23%], *I^2^* = 13%), the pooled body weight change was -6.76 kg (95% CI [-10.81 kg, -2.72 kg], *I^2^* = 99%); the amount of between-study heterogeneity of body weight was much increased to 20.84 with a wide 95% CI due to the limited included articles. However, the pooled correlation coefficient was positive of 0.81.** The pooled results for all participants and the results restricted to patients with diabetes are shown in **Fig 3.**

**Table 2 The pooled results for glycated hemoglobin level and weight reduction on comparison of glucagon-like peptide-1 receptor agonist and placebo by using the structural equation modeling multivariate meta-analysis according to participant characteristics**

|  | All participants | | | Patients with diabetes | | | Patients without diabetes | | |
| --- | --- | --- | --- | --- | --- | --- | --- | --- | --- |
| Setting *r* = 0.2 | Estimates (95% CI) | *p* | *I^2^* | Estimates (95% CI) | *p* | *I^2^* | Estimates (95% CI) | *p* | *I^2^* |
| Glycated hemoglobin (%) | -0.85 (-1.03, -0.66) | <0.001 | 99 % | -0.96 (-1.14, -0.79) | <0.001 | 96 % | -0.27 (-0.31, -0.23) | <0.001 | 13 % |
| Body weight (kg) | -4.03 (-5.11, -2.95) | <0.001 | 99 % | -3.23 (-3.86, -2.59) | <0.001 | 95 % | -6.76 (-10.81, -2.72) | 0.001 | 99 % |
| tao of glycated hemoglobin | 0.18 (0.07, 0.30) | <0.001 |  | 0.13 (0.03, 0.23) | 0.008 |  | 0 | 0.909 |  |
| tao of body weight | 7.36 (3.10, 11.63) | <0.001 |  | 1.77 (0.56, 2.99) | 0.004 |  | 20.84 (-5.32, 47.00) | 0.118 |  |
| Covariance | -0.48 (-1.02, 0.05) | <0.001 |  | 0.15 (-0.12, 0.43) | 0.281 |  | 0.04 (-0.14, 0.21) | 0.682 |  |
| Standardized correlation coefficient | -0.42 |  |  | 0.32 |  |  | 0.81 |  |  |

CI, confidence interval; *r,* correlation coefficient between glycated hemoglobin and body weight changes within the study level; tao, the variance of effect measure

**Fig 3.** The pooled effect sizes and their confidence ellipse for changes in glycated hemoglobin level and body weight in patients treated with a glucagon-like peptide-1 receptor agonist and placebo, in participants not restricted and restricted to diabetes.

(left) The pooled effect sizes and their confidence ellipse of the changes in the glycated hemoglobin level and body weight, in a comparison of glucagon-like peptide-1 receptor agonists and placebo in **all participants.**

(middle) The pooled effect sizes and their confidence ellipse for changes in the glycated hemoglobin level and body weight, in a comparison of glucagon-like peptide-1 receptor agonists and placebo in patients **with diabetes**.

(right) The pooled effect sizes and their confidence ellipse for changes in the glycated hemoglobin level and body weight, in a comparison of glucagon-like peptide-1 receptor agonists and placebo in patients **without diabetes**.

x-axis: Effects of glucagon-like peptide-1 receptor agonists on glycated hemoglobin level; y-axis: effects of glucagon-like peptide-1 receptor agonists on body weight changes; black dots: individual studies; ellipses with dashed lines: 95% confidence interval; red diamond: pooled effect and 95% confidence interval; smaller gray ellipse: 95% confidence interval; larger red ellipse: 95% prediction interval.

Quality assessment of primary studies to evaluate the reliability of study results is an essential and mandatory part of meta-analyses. Which scale was used for present study to asses the quality ? Kindly provide score of the studies in table 3.

**Response:** Thanks for your advice. In line 83: All included trials were assessed for bias using the **Cochrane risk-of-bias tool 2.0.**[14]

Reference #14.	Higgins JP, Thomas J, Chandler J, Cumpston M, Li T, Page MJ. Cochrane Handbook for Systematic Reviews of Interventions version 6.2: Cochrane; 2021.

We put the long table with 31 reference included articles in the **S3 Table**.

**S3 Table. Summary of risk of bias assessment for included studies.**

| **Study** | **Randomization process** | **Deviations from intended interventions** | **Missing outcome data** | **Measurement of the outcome** | **Selection of the reported result** | **Overall** |
| --- | --- | --- | --- | --- | --- | --- |
| Davies, 2021[1] | Low | Low | Low | Low | Low | Low |
| Gudbergsen, 2021[2] | Low | Low | Low | Low | Low | Low |
| Nealand, 2021[3] | Low | Low | Some concerns* | Low | Low | Some concerns |
| Wadden, 2021[4] | Low | Low | Low | Low | Low | Low |
| Wilding, 2021[5] | Low | Low | Low | Low | Low | Low |
| Garvey, 2020[6] | Low | Low | Low | Low | Low | Low |
| Ghanim, 2020[7] | Low | Low | Some concerns* | Low | Low | Some concerns |
| Aroda, 2019[8] | Low | Low | Low | Low | Low | Low |
| Gerstein, 2019[9] | Low | Low | Low | Low | Low | Low |
| Husain, 2019[10] | Low | Low | Low | Low | Low | Low |
| Mosenzonl, 2019[11] | Low | Low | Low | Low | Low | Low |
| van Eyk, 2019[12] | Low | Low | Low | Low | Low | Low |
| Zinman, 2019 (sc)[13] | Low | Low | Low | Low | Low | Low |
| Zinman, 2019 (oral)[14] | Low | Low | Low | Low | Low | Low |
| Frøssing, 2018[15] | Low | Low | Low | Low | Low | Low |
| Rodbard, 2018[16] | Low | Low | Low | Low | Low | Low |
| Ishøy, 2017[17] | Low | Low | Some concerns* | Low | Low | Some concerns |
| Mensberg, 2017[18] | Low | Low | Some concerns* | Low | Low | Some concerns |
| Sorli, 2017[19] | Low | Low | Low | Low | Low | Low |
| Blackman, 2016[20] | Low | Low | Low | Low | Low | Low |
| Davies, 2016[21] | Low | Low | Low | Low | Low | Low |
| Dejgaard, 2016[22] | Low | Low | Low | Low | Low | Low |
| Kuhadiya, 2016[23] | Low | Low | Low | Low | Low | Low |
| Marso, 2016[24] | Low | Low | Low | Low | Low | Low |
| Nauck, 2016[25] | Low | Low | Low | Low | Low | Low |
| Vanderheiden, 2016[26] | Low | Low | Low | Low | Low | Low |
| Ahmann, 2015[27] | Low | Low | Low | Low | Low | Low |
| Davies, 2015[28] | Low | Low | Low | Low | Low | Low |
| Retnakaran, 2014[29] | Low | Low | Low | Low | Low | Low |
| Kim, 2013[30] | Low | Low | Some concerns* | Low | Low | Some concerns |
| Wadden, 2013[31] | Low | Low | Low | Low | Low | Low |

* Not intention-to-treat analysis

1.	Gabery S, Salinas CG, Paulsen SJ, Ahnfelt-Rønne J, Alanentalo T, Baquero AF, et al. Semaglutide lowers body weight in rodents via distributed neural pathways. JCI Insight. 2020;5(6):e133429. doi: 10.1172/jci.insight.133429. PubMed PMID: 32213703.

2.	Secher A, Jelsing J, Baquero AF, Hecksher-Sørensen J, Cowley MA, Dalbøge LS, et al. The arcuate nucleus mediates GLP-1 receptor agonist liraglutide-dependent weight loss. The Journal of clinical investigation. 2014;124(10):4473-88. Epub 2014/09/10. doi: 10.1172/jci75276. PubMed PMID: 25202980; PubMed Central PMCID: PMCPMC4215190.

3.	Li J, He K, Ge J, Li C, Jing Z. Efficacy and safety of the glucagon-like peptide-1 receptor agonist oral semaglutide in patients with type 2 diabetes mellitus: A systematic review and meta-analysis. Diabetes research and clinical practice. 2021;172:108656. Epub 2021/01/13. doi: 10.1016/j.diabres.2021.108656. PubMed PMID: 33434602.

4.	Andreadis P, Karagiannis T, Malandris K, Avgerinos I, Liakos A, Manolopoulos A, et al. Semaglutide for type 2 diabetes mellitus: A systematic review and meta-analysis. Diabetes, obesity & metabolism. 2018;20(9):2255-63. Epub 2018/05/15. doi: 10.1111/dom.13361. PubMed PMID: 29756388.

5.	Avgerinos I, Michailidis T, Liakos A, Karagiannis T, Matthews DR, Tsapas A, et al. Oral semaglutide for type 2 diabetes: A systematic review and meta-analysis. Diabetes, obesity & metabolism. 2020;22(3):335-45. Epub 2019/10/23. doi: 10.1111/dom.13899. PubMed PMID: 31637820.

6.	Kristensen SL, Rørth R, Jhund PS, Docherty KF, Sattar N, Preiss D, et al. Cardiovascular, mortality, and kidney outcomes with GLP-1 receptor agonists in patients with type 2 diabetes: a systematic review and meta-analysis of cardiovascular outcome trials. The lancet Diabetes & endocrinology. 2019;7(10):776-85. Epub 2019/08/20. doi: 10.1016/s2213-8587(19)30249-9. PubMed PMID: 31422062.

7.	Bethel MA, Patel RA, Merrill P, Lokhnygina Y, Buse JB, Mentz RJ, et al. Cardiovascular outcomes with glucagon-like peptide-1 receptor agonists in patients with type 2 diabetes: a meta-analysis. The lancet Diabetes & endocrinology. 2018;6(2):105-13. Epub 2017/12/10. doi: 10.1016/s2213-8587(17)30412-6. PubMed PMID: 29221659.

8.	Davies M, Færch L, Jeppesen OK, Pakseresht A, Pedersen SD, Perreault L, et al. Semaglutide 2·4 mg once a week in adults with overweight or obesity, and type 2 diabetes (STEP 2): a randomised, double-blind, double-dummy, placebo-controlled, phase 3 trial. The Lancet. 2021;397(10278):971-84. doi: 10.1016/S0140-6736(21)00213-0.

9.	Neeland IJ, Marso SP, Ayers CR, Lewis B, Oslica R, Francis W, et al. Effects of liraglutide on visceral and ectopic fat in adults with overweight and obesity at high cardiovascular risk: a randomised, double-blind, placebo-controlled, clinical trial. The lancet Diabetes & endocrinology. 2021;9(9):595-605. Epub 2021/08/07. doi: 10.1016/s2213-8587(21)00179-0. PubMed PMID: 34358471.

10.	Gudbergsen H, Overgaard A, Henriksen M, Wæhrens EE, Bliddal H, Christensen R, et al. Liraglutide after diet-induced weight loss for pain and weight control in knee osteoarthritis: a randomized controlled trial. The American journal of clinical nutrition. 2021;113(2):314-23. Epub 2021/01/21. doi: 10.1093/ajcn/nqaa328. PubMed PMID: 33471039.

11.	Wadden TA, Bailey TS, Billings LK, Davies M, Frias JP, Koroleva A, et al. Effect of Subcutaneous Semaglutide vs Placebo as an Adjunct to Intensive Behavioral Therapy on Body Weight in Adults With Overweight or Obesity: The STEP 3 Randomized Clinical Trial. Jama. 2021;325(14):1403-13. Epub 2021/02/25. doi: 10.1001/jama.2021.1831. PubMed PMID: 33625476; PubMed Central PMCID: PMCPMC7905697 Nordisk (from grant support to the University of Pennsylvania) and personal fees from Novo Nordisk for service on a scientific advisory board during the conduct of the study, as well as personal fees from WW International (formerly Weight Watchers) for service on a scientific advisory board outside the submitted work. Dr Bailey reports receiving grants, personal fees, and nonfinancial support (writing assistance) from Novo Nordisk during the conduct of the study. Dr Billings reports receiving personal fees from Novo Nordisk, Sanofi, and Eli Lilly outside the submitted work. Dr Davies is co-funded by the NIHR Leicester Biomedical Research Centre and reports receiving consulting fees from Novo Nordisk; advisory board member, speaker, and consulting fees from Sanofi-Aventis, Eli Lilly, Merck Sharp & Dohme, Boehringer Ingelheim, AstraZeneca, and Janssen; advisory board fees from Lexicon, Servier, and Gilead Sciences Ltd; speaker fees from Napp Pharmaceuticals; and grants from Novo Nordisk, Sanofi-Aventis, Lilly, Boehringer Ingelheim, AstraZeneca, and Janssen outside the submitted work. Dr Frias reports receiving research support grants from Novo Nordisk during the conduct of the study; grants and personal fees from Boehringer Ingelheim, Eli Lilly, Merck, Novo Nordisk, and Sanofi; and grants from Janssen and Pfizer outside the submitted work. Dr Koroleva reports being an employee of Novo Nordisk A/S and holding shares in the company. Dr Lingvay reports receiving advisory board fees and consulting fees from AstraZeneca, consulting fees from Bayer HealthCare Pharmaceuticals, Eli Lilly and Company, Intarcia, Intercept Pharmaceuticals, Janssen Global Services, MannKind Corporation, Target Pharma, Valeritas, and Zealand Pharma; advisory board fees from Boehringer Ingelheim and Sanofi US Services; grant support, paid to UT Southwestern, from Merck; grant support, paid to her institution, from Mylan Pharmaceuticals and Pfizer; grant support, paid to UT Southwestern; and advisory board fees, consulting fees, and travel support from Novo Nordisk. Dr O'Neil reports receiving grants from Novo Nordisk during the conduct of the study; grants from Epitomee Medical, Eli Lilly, and WW International; and personal fees from Robard, Gedeon Richter, WebMD, and Novo Nordisk outside the submitted work. Dr Rubino reports receiving writing assistance from Novo Nordisk during the conduct of the study; serving as a clinical investigator for AstraZeneca, Boehringer Ingelheim, and Novo Nordisk; and reports owning Novo Nordisk shares of stock outside the submitted work. Dr Skovgaard reports spousal employment at Novo Nordisk. Dr Wallenstein reports receiving personal fees from Novo Nordisk during the conduct of the study and outside the submitted work. Dr Garvey reports receiving grants from Novo Nordisk; serving as site principal investigator for the clinical trial, which was sponsored by his university during the conduct of the study; receiving grants from Lexicon and Pfizer outside the submitted work; and serving as an ad hoc consultant on advisory committees for Jazz Pharmaceuticals, Boehringer Ingelheim, Novo Nordisk, and Pfizer. In each instance, he received no financial compensation, nor was there a financial relationship. No other disclosures were reported.

12.	Wilding JPH, Batterham RL, Calanna S, Davies M, Van Gaal LF, Lingvay I, et al. Once-Weekly Semaglutide in Adults with Overweight or Obesity. 2021;384(11):989-1002. doi: 10.1056/NEJMoa2032183. PubMed PMID: 33567185.

13.	Garvey WT, Birkenfeld AL, Dicker D, Mingrone G, Pedersen SD, Satylganova A, et al. Efficacy and Safety of Liraglutide 3.0 mg in Individuals With Overweight or Obesity and Type 2 Diabetes Treated With Basal Insulin: The SCALE Insulin Randomized Controlled Trial. Diabetes care. 2020;43(5):1085-93. Epub 2020/03/07. doi: 10.2337/dc19-1745. PubMed PMID: 32139381; PubMed Central PMCID: PMCPMC7171937.

14.	Ghanim H, Batra M, Green K, Abuaysheh S, Hejna J, Makdissi A, et al. Liraglutide treatment in overweight and obese patients with type 1 diabetes: A 26-week randomized controlled trial; mechanisms of weight loss. Diabetes, obesity & metabolism. 2020;22(10):1742-52. Epub 2020/05/20. doi: 10.1111/dom.14090. PubMed PMID: 32424935.

15.	Aroda VR, Rosenstock J, Terauchi Y, Altuntas Y, Lalic NM, Morales Villegas EC, et al. PIONEER 1: Randomized Clinical Trial of the Efficacy and Safety of Oral Semaglutide Monotherapy in Comparison With Placebo in Patients With Type 2 Diabetes. Diabetes care. 2019;42(9):1724-32. Epub 2019/06/13. doi: 10.2337/dc19-0749. PubMed PMID: 31186300.

16.	Gerstein HC, Colhoun HM, Dagenais GR, Diaz R, Lakshmanan M, Pais P, et al. Dulaglutide and cardiovascular outcomes in type 2 diabetes (REWIND): a double-blind, randomised placebo-controlled trial. Lancet (London, England). 2019;394(10193):121-30. Epub 2019/06/14. doi: 10.1016/s0140-6736(19)31149-3. PubMed PMID: 31189511.

17.	Husain M, Birkenfeld AL, Donsmark M, Dungan K, Eliaschewitz FG, Franco DR, et al. Oral Semaglutide and Cardiovascular Outcomes in Patients with Type 2 Diabetes. The New England journal of medicine. 2019;381(9):841-51. Epub 2019/06/12. doi: 10.1056/NEJMoa1901118. PubMed PMID: 31185157.

18.	Mosenzon O, Blicher TM, Rosenlund S, Eriksson JW, Heller S, Hels OH, et al. Efficacy and safety of oral semaglutide in patients with type 2 diabetes and moderate renal impairment (PIONEER 5): a placebo-controlled, randomised, phase 3a trial. The lancet Diabetes & endocrinology. 2019;7(7):515-27. Epub 2019/06/14. doi: 10.1016/s2213-8587(19)30192-5. PubMed PMID: 31189517.

19.	van Eyk HJ, Paiman EHM, Bizino MB, de Heer P, Geelhoed-Duijvestijn PH, Kharagjitsingh AV, et al. A double-blind, placebo-controlled, randomised trial to assess the effect of liraglutide on ectopic fat accumulation in South Asian type 2 diabetes patients. Cardiovascular diabetology. 2019;18(1):87. Epub 2019/07/11. doi: 10.1186/s12933-019-0890-5. PubMed PMID: 31288820; PubMed Central PMCID: PMCPMC6615254.

20.	Zinman B, Bhosekar V, Busch R, Holst I, Ludvik B, Thielke D, et al. Semaglutide once weekly as add-on to SGLT-2 inhibitor therapy in type 2 diabetes (SUSTAIN 9): a randomised, placebo-controlled trial. The lancet Diabetes & endocrinology. 2019;7(5):356-67. Epub 2019/03/06. doi: 10.1016/s2213-8587(19)30066-x. PubMed PMID: 30833170.

21.	Zinman B, Aroda VR, Buse JB, Cariou B, Harris SB, Hoff ST, et al. Efficacy, Safety, and Tolerability of Oral Semaglutide Versus Placebo Added to Insulin With or Without Metformin in Patients With Type 2 Diabetes: The PIONEER 8 Trial. Diabetes care. 2019;42(12):2262-71. Epub 2019/09/19. doi: 10.2337/dc19-0898. PubMed PMID: 31530667; PubMed Central PMCID: PMCPMC7364672.

22.	Frøssing S, Nylander M, Chabanova E, Frystyk J, Holst JJ, Kistorp C, et al. Effect of liraglutide on ectopic fat in polycystic ovary syndrome: A randomized clinical trial. Diabetes, obesity & metabolism. 2018;20(1):215-8. Epub 2017/07/07. doi: 10.1111/dom.13053. PubMed PMID: 28681988.

23.	Rodbard HW, Lingvay I, Reed J, de la Rosa R, Rose L, Sugimoto D, et al. Semaglutide Added to Basal Insulin in Type 2 Diabetes (SUSTAIN 5): A Randomized, Controlled Trial. The Journal of clinical endocrinology and metabolism. 2018;103(6):2291-301. Epub 2018/04/25. doi: 10.1210/jc.2018-00070. PubMed PMID: 29688502; PubMed Central PMCID: PMCPMC5991220.

24.	Ishøy PL, Knop FK, Broberg BV, Bak N, Andersen UB, Jørgensen NR, et al. Effect of GLP-1 receptor agonist treatment on body weight in obese antipsychotic-treated patients with schizophrenia: a randomized, placebo-controlled trial. Diabetes, Obesity and Metabolism. 2017;19(2):162-71. doi: 10.1111/dom.12795. PubMed Central PMCID: PMCAstra Zeneca(Sweden).

25.	Mensberg P, Nyby S, Jørgensen PG, Storgaard H, Jensen MT, Sivertsen J, et al. Near-normalization of glycaemic control with glucagon-like peptide-1 receptor agonist treatment combined with exercise in patients with type 2 diabetes. Diabetes, Obesity and Metabolism. 2017;19(2):172-80. doi: 10.1111/dom.12797. PubMed Central PMCID: PMCNovo Nordisk.

26.	Sorli C, Harashima SI, Tsoukas GM, Unger J, Karsbøl JD, Hansen T, et al. Efficacy and safety of once-weekly semaglutide monotherapy versus placebo in patients with type 2 diabetes (SUSTAIN 1): a double-blind, randomised, placebo-controlled, parallel-group, multinational, multicentre phase 3a trial. The lancet Diabetes & endocrinology. 2017;5(4):251-60. Epub 2017/01/24. doi: 10.1016/s2213-8587(17)30013-x. PubMed PMID: 28110911.

27.	Blackman A, Foster GD, Zammit G, Rosenberg R, Aronne L, Wadden T, et al. Effect of liraglutide 3.0 mg in individuals with obesity and moderate or severe obstructive sleep apnea: the SCALE Sleep Apnea randomized clinical trial. International journal of obesity (2005). 2016;40(8):1310-9. Epub 2016/03/24. doi: 10.1038/ijo.2016.52. PubMed PMID: 27005405; PubMed Central PMCID: PMCPMC4973216 Valeant Canada, Paladin Labs Inc. Dr Foster: at the time of the study was a scientific advisory board participant for Con Agra Foods, United Health Group and Tate and Lyle, and a consultant to Eisai and Novo Nordisk; currently employed by Weight Watchers International. Dr Zammit: consultant for Acorda, Actelion, Alexza, Arena, Aventis, Biovail, Boehringer-Ingelheim, Cephalon, Elan, Eli Lilly, Evotec, Forest, Glaxo Smith Kline, Jazz, King Pharmaceuticals, Ligand, McNeil, Merck, Neurocrine Biosciences, Organon, Pfizer, Purdue, Renovis, Sanofi-Aventis, Select Comfort, Sepracor, Shire, Somnus, Takeda Pharmaceuticals, Vela, Wyeth; grants/research support from Abbott, Abbvie, Actelion, Ancile, Apnex, Arena, Astra-Zeneca, Aventis, Banyu, Biomarin, BMS, Catalyst, Cephalon Inc., CHDI, Elan, Epix, Eisai, Elminda, Evotec, Forest, Galderma, Genentech, Gilead, Glaxo Smith Kline, Gilead, H. Lundbeck A/S, Janssen, Johnson & Johnson, King, Merck and Co., National Institute of Health (NIH), Neurim, Neurocrine Biosciences, Naurex, Neurim, Neurogen, Novo Nordisk, Organon, Orphan Medical, Otsuka, Pfizer, Predix, Respironics, Saladax, Sanofi-Aventis, Sanofi-Synthelabo, Schering-Plough, Sepracor, Sunovion, Shire, Somaxon, Takeda Pharmaceuticals North America, Targacept, Teva, Thymon, Transcept, UCB Pharma, Ultragenyx, Predix, Vanda, Wyeth-Ayerst Research; honoraria received from Neurocrine Biosciences, King Pharmaceuticals, McNeil, Sanofi-Aventis, Sanofi-Synthelabo, Sepracor, Takeda Pharmaceuticals, Vela Pharmaceuticals, Wyeth-Ayerst Research; ownership interest in Clinilabs, Inc., Clinilabs IPA, Inc., Home Sleep and Respiratory Care, Nationwide Sleep Testing. Dr Rosenberg: received research grant support from Merck, Novo Nordisk, Pfizer, Teva, Jazz Pharmaceuticals, Sunovion Pharmaceuticals and Philips-Respironics. Dr Aronne: advisory board member for Myos Corporation; consultant, speaker, advisor, or received research support from Aspire Bariatrics Inc., Eisai, Ethicon Endo-Surgery Inc., GlaxoSmithKline Consumer Healthcare LP, GI Dynamics, Novo Nordisk, Pfizer, Takeda Pharmaceuticals, Vivus Inc., Zafgen Inc.; ownership interest in Myos Corporation and BMIQ. Dr Wadden: advisory board participant for Orexigen Pharmaceuticals, Inc., Novo Nordisk, Nutrisystem, and Shire Pharmaceuticals, with research support (to the University of Pennsylvania) from the first three companies. Dr Claudius: employee of Novo Nordisk and owns company stock. Dr Jensen: employee of Novo Nordisk and owns company stock. Dr Mignot: advisory board member, consultant for and research support from Jazz Pharmaceuticals; consultant for and research grant from Novo Nordisk; consultant for Reset Pharmaceuticals and Merck; expert witness for FTC.

28.	Davies MJ, Bain SC, Atkin SL, Rossing P, Scott D, Shamkhalova MS, et al. Efficacy and Safety of Liraglutide Versus Placebo as Add-on to Glucose-Lowering Therapy in Patients With Type 2 Diabetes and Moderate Renal Impairment (LIRA-RENAL): A Randomized Clinical Trial. Diabetes care. 2016;39(2):222-30. Epub 2015/12/19. doi: 10.2337/dc14-2883. PubMed PMID: 26681713.

29.	Dejgaard TF, Frandsen CS, Hansen TS, Almdal T, Urhammer S, Pedersen-Bjergaard U, et al. Efficacy and safety of liraglutide for overweight adult patients with type 1 diabetes and insufficient glycaemic control (Lira-1): A randomised, double-blind, placebo-controlled trial. The Lancet Diabetes and Endocrinology. 2016;4(3):221-32. doi: 10.1016/S2213-8587(15)00436-2. PubMed Central PMCID: PMCNovo Nordisk(Denmark).

30.	Kuhadiya ND, Dhindsa S, Ghanim H, Mehta A, Makdissi A, Batra M, et al. Addition of Liraglutide to Insulin in Patients With Type 1 Diabetes: A Randomized Placebo-Controlled Clinical Trial of 12 Weeks. Diabetes care. 2016;39(6):1027-35. Epub 2016/05/22. doi: 10.2337/dc15-1136. PubMed PMID: 27208343; PubMed Central PMCID: PMCPMC5864130.

31.	Marso SP, Bain SC, Consoli A, Eliaschewitz FG, Jódar E, Leiter LA, et al. Semaglutide and Cardiovascular Outcomes in Patients with Type 2 Diabetes. 2016;375(19):1834-44. doi: 10.1056/NEJMoa1607141. PubMed PMID: 27633186.

32.	Nauck MA, Stewart MW, Perkins C, Jones-Leone A, Yang F, Perry C, et al. Efficacy and safety of once-weekly GLP-1 receptor agonist albiglutide (HARMONY 2): 52 week primary endpoint results from a randomised, placebo-controlled trial in patients with type 2 diabetes mellitus inadequately controlled with diet and exercise. Diabetologia. 2016;59(2):266-74. Epub 2015/11/19. doi: 10.1007/s00125-015-3795-1. PubMed PMID: 26577795; PubMed Central PMCID: PMCPMC4705137.

33.	Vanderheiden A, Harrison L, Warshauer J, Li X, Adams-Huet B, Lingvay I. Effect of Adding Liraglutide vs Placebo to a High-Dose lnsulin Regimen in Patients With Type 2 Diabetes: A Randomized Clinical Trial. JAMA Internal Medicine. 2016;176(7):939-47. doi: 10.1001/jamainternmed.2016.1540 %J JAMA Internal Medicine.

34.	Ahmann A, Rodbard HW, Rosenstock J, Lahtela JT, de Loredo L, Tornøe K, et al. Efficacy and safety of liraglutide versus placebo added to basal insulin analogues (with or without metformin) in patients with type 2 diabetes: a randomized, placebo-controlled trial. Diabetes, obesity & metabolism. 2015;17(11):1056-64. Epub 2015/07/17. doi: 10.1111/dom.12539. PubMed PMID: 26179619; PubMed Central PMCID: PMCPMC5054929.

35.	Davies MJ, Bergenstal R, Bode B, Kushner RF, Lewin A, Skjøth TV, et al. Efficacy of Liraglutide for Weight Loss Among Patients With Type 2 Diabetes: The SCALE Diabetes Randomized Clinical Trial. Jama. 2015;314(7):687-99. Epub 2015/08/19. doi: 10.1001/jama.2015.9676. PubMed PMID: 26284720.

36.	Retnakaran R, Kramer CK, Choi H, Swaminathan B, Zinman B. Liraglutide and the preservation of pancreatic β-cell function in early type 2 diabetes: the LIBRA trial. Diabetes care. 2014;37(12):3270-8. Epub 2014/09/25. doi: 10.2337/dc14-0893. PubMed PMID: 25249651.

37.	Kim SH, Abbasi F, Lamendola C, Liu A, Ariel D, Schaaf P, et al. Benefits of liraglutide treatment in overweight and obese older individuals with prediabetes. Diabetes care. 2013;36(10):3276-82. Epub 2013/07/10. doi: 10.2337/dc13-0354. PubMed PMID: 23835684; PubMed Central PMCID: PMCPMC3781545.

38.	Wadden TA, Hollander P, Klein S, Niswender K, Woo V, Hale PM, et al. Weight maintenance and additional weight loss with liraglutide after low-calorie-diet-induced weight loss: the SCALE Maintenance randomized study. International journal of obesity (2005). 2013;37(11):1443-51. Epub 2013/07/03. doi: 10.1038/ijo.2013.120. PubMed PMID: 23812094.

39.	Huthmacher JA, Meier JJ, Nauck MA. Efficacy and Safety of Short- and Long-Acting Glucagon-Like Peptide 1 Receptor Agonists on a Background of Basal Insulin in Type 2 Diabetes: A Meta-analysis. Diabetes care. 2020;43(9):2303-12. Epub 2020/09/11. doi: 10.2337/dc20-0498. PubMed PMID: 32910778.

**Reviewer #3:**

1. Title: Ambiguous- the authors need to modify the title. We suggest “Effect of glucagon-like peptide-1 receptor agonists on glycemic control, and weight reduction in adults: a multivariate meta-analysis”

**Response:** Thanks for your advice. We modified the title to “**Effect of glucagon-like peptide-1 receptor agonists on glycemic control, and weight reduction in adults: a multivariate meta-analysis**”.

2. Abstract

a) Aims: Requires modification to be in consonance with the new title

**Response:** Thanks for your advice. We modified the aims of the **abstract**:

**Previous** aims of the abstract: ~~To explore the effect of glucagon-like peptide-1 receptor agonist (GLP-1 Ras) on glycemic control and weight reduction in adults.~~

**Revised** aims of the abstract: **To explore the effect of glucagon-like peptide-1 receptor agonist (GLP-1 Ras) on glycemic control and weight reduction in adults.**

b) Methods: First sentence- The authors should state the exact time of commencement of the study and not just state “from study inception”.

**Response:** Thanks for your advice. We modified the methods of the abstract: **Databases were searched from ~~study inception~~** **August 2021 to March 2022.**

c) Conclusion: The last sentence should be moved to the results section. However, the implication(s) of the result contained in the sentence can be included in the conclusion section as done in the conclusion section of the manuscript.

**Response:** Thanks for your advice. The last sentence of the manuscript was moved to the results section.

Original abstract:

Results. ….The standardized pooled correlation coefficient between HbA1c levels and body weight was -0.40.

Conclusion. Long-acting GLP-1 RAs significantly reduced the glycated hemoglobin level and body weight in adults. **However, a negative correlation between glycemic control and weight reduction was obtained**.

Revised abstract:

Results. ….The standardized pooled correlation coefficient between HbA1c levels and body weight was -0.40. **A negative correlation between glycemic control and weight reduction was obtained.**

Conclusion. Long-acting GLP-1 RAs significantly reduced the glycated hemoglobin level and body weight in adults. **~~However, a negative correlation between glycemic control and weight reduction was obtained~~**~~.~~

Original conclusion of the **manuscript**:

In conclusion, long-acting GLP-1 RAs significantly lowered HbA1c levels and body weight in adults. However, the positive association between glycemic control and weight reduction **~~in diabetic patients could not be expected in non-diabetic patients treated with long-acting GLP-1 RAs.~~**

Revised conclusion of the **manuscript**:

In conclusion, long-acting GLP-1 RAs significantly lowered HbA1c levels and body weight in adults. However, the positive association between glycemic control and weight reduction **was only observed in diabetic patients and in non-diabetic participants, but not in all participants with high heterogeneity treated with long-acting GLP-1 RAs.**

3. Introduction: Well written

4. Materials and Methods:

a) Line 61: There is the need for the authors to define the word “inception” and state categorically the date the study commenced OR did the authors mean since the inception of the databases? If yes, this should be expressly stated.

**Response:** Thanks for your advice. We revised the methos:

Original Search strategy and selection criteria

We searched the Medline, Ovid EMBASE, Cochrane Library and ClinicalTrials.gov databases for relevant studies **from inception** to March 2022 by using the following keywords:

Revised Search strategy and selection criteria

We searched the Medline, Ovid EMBASE, Cochrane Library and ClinicalTrials.gov databases for relevant studies **from ~~inception~~** **August 2021** to March 2022 by using the following keywords:

b) Lines 83 – 85: The last sentence in the methodology section should be moved to appropriate place as it described specific roles of some authors.

**Response:** Thanks for your advice. We deleted the last sentence in the methodology section: All included trials were assessed for bias using the Cochrane risk-of-bias tool 2.0.[14] **~~The authors M. C. T. and W. H. T. conducted the searches and conducted quality assessments independently, and disagreements were resolved through consensus.~~**

5. Data analysis: This section is too long. I would suggest that the authors revise lines 88 – 99 or move it to the supplementary section..

**Response:** Thanks for your advice. We move lines 88 – 99 to the supplementary section.

Revised methods:…All included trials were assessed for bias using the Cochrane risk-of-bias tool 2.0.[14]. **The details of the data extraction in our study were described in supplement (S1 File).**

**S1 File. Details of data extraction in the study:** The outcomes corresponding to glycemic changes were usually differences in HbA1c levels, changes in fasting plasma glucose levels, changes in self-monitored blood glucose levels from baseline, differences between groups, rate of achievement of an HbA1c target of 6.5% or 7.0%, or other measurements of insulin level or homeostatic model assessment scores among different studies. The outcomes of anthropometric changes were usually differences in body weight, body mass index (BMI), or waist circumference from baseline, differences between groups, or the rate of achievement of a 5%–10% reduction in body weight or BMI. To avoid unit-of-analysis errors,[15] we only extracted the most commonly reported, estimated treatment difference in HbA1c level and body weight from the baseline corresponding to a full dose of GLP-1 RAs in each RCT. The estimated treatment difference was determined using the Revman calculator with the numbers in each arm and the p-values.

6. Results:

a) Table 1: The authors should unbold the results in bold format. The contents of the Table are already explained in lines 155 – 159 and the p-values are self-explanatory.

**Response:** Thanks for your advice. We unbold the results in bold format.

b) There is the need to ensure uniform decimal points for the p-values.

**Response:** Thanks for your advice. We uniformed the decimal points for the p-values.

c) The authors should remove the sentence “bold indicates statistical significance” from the legend since the results will be unbolded.

**Response:** Thanks for your advice. We remove the sentence “bold indicates statistical significance” from the legend

d) Going through row 3 in Table 1, Age vs Body weight has p-value of 0.072 which is not significant but was bolded.

**Response:** Thanks for your advice. We unbold the results in Tables now.

The **original** **Table 1 Univariate meta-regression of the effects of glucagon-like peptide-1 receptor agonists on glycated hemoglobin levels and weight reduction**

|  | Glycated hemoglobin | | | Body weight | | |
| --- | --- | --- | --- | --- | --- | --- |
|  | MD (95% CI) | *p* | R^2^(%) | MD (95% CI) | *p* | R^2^(%) |
| Age (years) | **-0.027 (-0.050, -0.003)** | **0.027** | **18.7** | 0.100 (-0.010, 0.210) | **0.072** | 7.0 |
| Proportion of women (%) | **0.016 (0.004, 0.028)** | **0.011** | **20.4** | **-0.070 (-0.121, -0.019)** | **0.009** | **20.3** |
| Baseline glycated hemoglobin (%) | **-0.255 (-0.485, -0.026)** | **0.031** | **22.0** | 0.818 (-0.440, 2.075) | 0.192 | 3.0 |
| Baseline body mass index (kg/m^2^) | 0.028 (-0.040, 0.096) | 0.40 | 0.2 | -0.258 (-0.584, 0.068) | 0.12 | 6.6 |
| Duration of diabetes (years) | -0.002 (-0.033, 0.030) | 0.92 | 0 | 0.084 (-0.082, 0.250) | 0.31 | 1.0 |
| Study duration (weeks) | 0.002 (-0.007, 0.011) | 0.70 | 0 | **-0.049 (-0.092, -0.006)** | **0.028** | **13.1** |

CI, confidence interval; MD, mean difference**;** R^2^ (%), percentage of heterogeneity explained; **bold indicates statistical significance**

The **revised** **Table 1 Univariate meta-regression of the effects of glucagon-like peptide-1 receptor agonists on glycated hemoglobin levels and weight reduction**

|  | Glycated hemoglobin | | | Body weight | | |
| --- | --- | --- | --- | --- | --- | --- |
|  | MD (95% CI) | *p* | R^2^(%) | MD (95% CI) | *p* | R^2^(%) |
| Age (years) | -0.026 (-0.049, -0.002) | 0.032 | 17.2 | 0.093 (-0.016, 0.202) | 0.091 | 6.0 |
| Proportion of women (%) | 0.015 (0.003, 0.027) | 0.017 | 17.3 | -0.065 (-0.117, -0.013) | 0.016 | 18.0 |
| Baseline glycated hemoglobin (%) | -0.262 (-0.473, -0.050) | 0.018 | 25.1 | 1.130 (-0.132, 2.393) | 0.077 | 9.6 |
| Baseline body mass index (kg/m^2^) | 0.021 (-0.045, 0.087) | 0.514 | 0 | -0.217 (-0.553, 0.120) | 0.197 | 3.5 |
| Baseline body weight (kg) | 0.010 (-0.013, 0.033) | 0.374 | 1.8 | -0.045 (-0.157, 0.068) | 0.424 | 0 |
| Duration of diabetes (years) | -0.004 (-0.035, 0.027) | 0.788 | 0 | 0.105 (-0.060, 0.269) | 0.203 | 3.7 |
| Study duration (weeks) | 0.002 (-0.007, 0.011) | 0.660 | 0 | -0.052 (-0.094, -0.010) | 0.018 | 16.2 |
| Gastrointestinal side effect (%) | 0.017 (0.007, 0.027) | 0.002 | 35.1 | -0.059 (-0.110, -0.008) | 0.025 | 15.1 |
| Insulin use (%) | 0.000 (-0.005, 0.005) | 0.863 | 0 | 0.015 (-0.011, 0.040) | 0.249 | 2.2 |

CI, confidence interval; MD, mean difference**;** R^2^ (%), percentage of heterogeneity explained**~~; bold indicates statistical significance~~**

7. Discussion:

a) Line 263- Long-acting GLP-1 Ras “showed” and not “show”.

**Response:** Thanks for your advice. We revised the sentence: **Long-acting GLP-1 RAs showed better efficacy in weight reduction and glycemic control than short-acting GLP-1 RAs.[39]**

8. Conclusion: Sentence 2- Replace “could” with “might”

**Response:** Thanks for your advice. We revised the discussion: Our meta-analysis demonstrated that long-acting GLP-1 RAs significantly reduced HbA1c levels and body weight in adults. **The high heterogeneity in our study ~~could~~ might be attributed to the different GLP-1 RAs and the diverse populations ranging from non-diabetic overweight/obese participants to patients with diabetes complicated with end organ damage.**

Other Comments

1. Inclusion of withdrawn, terminated and unknown status studies as stated in lines 278 – 280 is a source of concern as they could obscure the true findings and conclusion of this study. Is it possible for the authors to reanalyze the retrieved data without the aforementioned studies and find out if a similar outcome will still emerge?

**Response:** Thanks for your advice. We **did not analyze** the articles in lines 278 – 280, the withdrawn, terminated and unknown status studies. We mentioned the above studies in the discussion due to these studies are **potential unpublished studies, the possible source of the publication bias.**

2. Did the authors exclude studies that included type 2 diabetics on insulin in the multivariate analysis as this could affect both the glycemic control and body weight?

**Response:** Thanks for your advice. We did **not** **exclude** studies that included type 2 diabetics on insulin in the multivariate analysis, thus insulin could affect both the glycemic control and body weight. We **performed meta-regression with insulin with the outcomes:**

The revised **results**:

Meta-regression (**Table 1**) showed that the pooled **HbA1c reduction** significantly interacted with participants’ baseline age (*p* = 0.032), proportion of female participants (*p* = 0.017), the baseline HbA1c level (*p* = 0.018), and gastrointestinal side effect (*p* = 0.002) but **did not interact with** baseline body weight or BMI level, duration of diabetes, follow-up period or **insulin use**….

Meta-regression (**Table 1**) showed that the pooled **body weight** reduction significantly interacted with the proportion of female participants (*p* = 0.016), the follow-up period (*p* = 0.018) and gastrointestinal side effect (*p* = 0.025), and had a borderline interaction with participants’ baseline age (*p* = 0.091) and the participants’ baseline HbA1c level (*p* = 0.077), but **did not interact with** baseline BMI level or body weight, **insulin use,** or the duration of diabetes….

The revised Table 1 **Univariate meta-regression of the effects of glucagon-like peptide-1 receptor agonists on glycated hemoglobin levels and weight reduction**

|  | Glycated hemoglobin | | | Body weight | | |
| --- | --- | --- | --- | --- | --- | --- |
|  | MD (95% CI) | *p* | R^2^(%) | MD (95% CI) | *p* | R^2^(%) |
| Age (years) | -0.026 (-0.049, -0.002) | 0.032 | 17.2 | 0.093 (-0.016, 0.202) | 0.091 | 6.0 |
| Proportion of women (%) | 0.015 (0.003, 0.027) | 0.017 | 17.3 | -0.065 (-0.117, -0.013) | 0.016 | 18.0 |
| Baseline glycated hemoglobin (%) | -0.262 (-0.473, -0.050) | 0.018 | 25.1 | 1.130 (-0.132, 2.393) | 0.077 | 9.6 |
| Baseline body mass index (kg/m^2^) | 0.021 (-0.045, 0.087) | 0.514 | 0 | -0.217 (-0.553, 0.120) | 0.197 | 3.5 |
| Baseline body weight (kg) | 0.010 (-0.013, 0.033) | 0.374 | 1.8 | -0.045 (-0.157, 0.068) | 0.424 | 0 |
| Duration of diabetes (years) | -0.004 (-0.035, 0.027) | 0.788 | 0 | 0.105 (-0.060, 0.269) | 0.203 | 3.7 |
| Study duration (weeks) | 0.002 (-0.007, 0.011) | 0.660 | 0 | -0.052 (-0.094, -0.010) | 0.018 | 16.2 |
| Gastrointestinal side effect (%) | 0.017 (0.007, 0.027) | 0.002 | 35.1 | -0.059 (-0.110, -0.008) | 0.025 | 15.1 |
| Insulin use (%) | 0.000 (-0.005, 0.005) | 0.863 | 0 | 0.015 (-0.011, 0.040) | 0.249 | 2.2 |

CI, confidence interval; MD, mean difference**;** R^2^ (%), percentage of heterogeneity explained

3. The observed negative correlation between glycemic control and weight reduction in the univariate analysis is not unexpected because of the diverse population of the participants, different types of GLP-1 RAs and the publication bias.

Suggestion

This manuscript might benefit from a Biostatistician input(s) as I have limited knowledge on the statistical tools/methods used in the study.

**Response:** Thanks for your advice. The manuscript was supervised under a chief biostatistician professor in Institute of Epidemiology and Preventive Medicine, College of Public Health, National Taiwan University, Taipei, Taiwan

6. PLOS authors have the option to publish the peer review history of their article (what does this mean?). If published, this will include your full peer review and any attached files.

If you choose “no”, your identity will remain anonymous but your review may still be made public.

Do you want your identity to be public for this peer review? For information about this choice, including consent withdrawal, please see our Privacy Policy.

Reviewer #1: Yes: Yuichiro Adachi

Reviewer #2: No

Reviewer #3: No
